# Mixotrophic growth of the extremophile *Galdieria sulphuraria* reveals the flexibility of its carbon assimilation metabolism

**DOI:** 10.1111/nph.17359

**Published:** 2021-05-01

**Authors:** Gilles Curien, Dagmar Lyska, Erika Guglielmino, Phillip Westhoff, Janina Janetzko, Marianne Tardif, Clément Hallopeau, Sabine Brugière, Davide Dal Bo, Johan Decelle, Benoit Gallet, Denis Falconet, Michele Carone, Claire Remacle, Myriam Ferro, Andreas P.M. Weber, Giovanni Finazzi

**Affiliations:** ^1^ Laboratoire de Physiologie Cellulaire et Végétale. Université Grenoble Alpes CNRS CEA INRAe Grenoble Cedex 9 38054 France; ^2^ Institute of Plant Biochemistry Cluster of Excellence on Plant Sciences (CEPLAS) Heinrich Heine University Düsseldorf 40225 Germany; ^3^ EdyP Laboratoire Biologie à Grande Echelle, Université Grenoble Alpes CEA Inserm BGE U1038 Grenoble Cedex 9 38054 France; ^4^ Institut de Biologie Structurale Université Grenoble Alpes CNRS CEA 71 Avenue des Martyrs Grenoble 38044 France; ^5^ Genetics and Physiology of Microalgae InBios/Phytosystems Research Unit University of Liege Liège 4000 Belgium

**Keywords:** *Galdieria sulphuraria*, mixotrophy, photorespiration, photosynthesis, red algae

## Abstract

- *Galdieria* *sulphuraria* is a cosmopolitan microalga found in volcanic hot springs and calderas. It grows at low pH in photoautotrophic (use of light as a source of energy) or heterotrophic (respiration as a source of energy) conditions, using an unusually broad range of organic carbon sources. Previous data suggested that *G*. *sulphuraria* cannot grow mixotrophically (simultaneously exploiting light and organic carbon as energy sources), its photosynthetic machinery being repressed by organic carbon.
- Here, we show that *G*. *sulphuraria* SAG21.92 thrives in photoautotrophy, heterotrophy and mixotrophy. By comparing growth, biomass production, photosynthetic and respiratory performances in these three trophic modes, we show that addition of organic carbon to cultures (mixotrophy) relieves inorganic carbon limitation of photosynthesis thanks to increased CO_2_ supply through respiration. This synergistic effect is lost when inorganic carbon limitation is artificially overcome by saturating photosynthesis with added external CO_2_.
- Proteomic and metabolic profiling corroborates this conclusion suggesting that mixotrophy is an opportunistic mechanism to increase intracellular CO_2_ concentration under physiological conditions, boosting photosynthesis by enhancing the carboxylation activity of Ribulose‐1,5‐bisphosphate carboxylase‐oxygenase (Rubisco) and decreasing photorespiration.
- We discuss possible implications of these findings for the ecological success of Galdieria in extreme environments and for biotechnological applications.

## Introduction

The unicellular red alga *Galdieria* *sulphuraria* belongs to the Cyanidiophyceae, a class that includes five species often flourishing in different extreme environments (Merola *et* *al.,* 1981; Gross *et* *al.,* 1998; Gross & Oesterhelt, 1999; Oesterhelt *et* *al.,* 2007). From a phylogenetic perspective, plastids of the red algae gave rise to the complex plastids of, e.g. diatoms via secondary endosymbiosis (Yoon *et* *al.,* 2002, 2004; Bhattacharya *et* *al.,* 2003). Like other members of this class (Doemel & Brock, 1971; Reeb & Bhattacharya, 2010) *G*. *sulphuraria* has an extremophile lifestyle, withstanding low pH (pH optimum at 2) and elevated temperatures (up to 56°C). It thrives in soils and forms biomats on rocks surrounding hot springs, fumaroles, or acid mining sites and even on burning coal spoil heaps (Moreira *et* *al.,* 1994; Gross *et* *al.,* 1998; Castenholz & McDermott, 2010; Barcyté *et* *al.,* 2018). Some mesophilic species have been isolated from environments with moderate temperatures and/or a neutral pH (Gross *et* *al.,* 2002; Yoon *et* *al.,* 2006; Azúa‐Bustos *et* *al.,* 2009; Iovinella *et* *al.,* 2018). Genome analysis (Barbier *et* *al.,* 2005; Schonknecht *et* *al.,* 2013; Rossoni *et* *al.,* 2019a) has pinpointed a very high metabolic flexibility of this alga, which is confirmed by its ability to grow in photoautotrophy (exclusive use of light as an energy source) and heterotrophy (organic carbon respiration of more than 50 different substrates (Gross & Schnarrenberger, 1995)). This capacity, along with the peculiar pH optimum for growth, allow *G*. *sulphuraria* to be cultivated in open ponds containing organic matter, overcoming other microorganisms, considered as contaminants in this case. Given these advantages for large‐scale cultivation, *G*. *sulphuraria* is considered an emerging system for biotechnology applications (Schmidt *et* *al.,* 2005; Henkanatte‐Gedera *et* *al.,* 2017; Cizkova *et* *al.,* 2019).

The relationship between photosynthesis and glycolysis/respiration in higher plants and microalgae is complex (Avelange *et* *al.,* 1988; Kromer *et* *al.,* 1988; Gemel & Randall, 1992; Pärnik & Keerberg, 1995; Hoefnagel *et* *al.,* 1998; Tcherkez *et* *al.,* 2008). Photosynthesis/glycolysis/respiration interactions are prone to perturbation by mixotrophy, in which external organic carbon often interferes with carbon flow between chloroplasts, the cytosol and mitochondria. Several green microalgae are capable of mixotrophic growth (Combres *et* *al.,* 1994; Wan *et* *al.,* 2011; Johnson & Alric, 2012; Cecchin *et* *al.,* 2018). While mixotrophy has always beneficial consequences on respiration, its effects on photosynthesis differ depending on the microalga considered: enhancement of photosynthesis was reported in one case (*Ettlia* *oleoabundans* (Ferroni *et* *al.,* 2018)), while in other algae, including the diatoms *Phaeodactylum* *tricornutum* (Liu *et* *al.,* 2009; Villanova *et* *al.,* 2017) and *Nannochloropsis* (Fang *et* *al.,* 2004; Xu *et* *al.,* 2004) photosynthesis was unaffected. Decreased photosynthetic activity in mixotrophy has been reported in *Chlorella* *vulgaris* (Martinez & Orus, 1991; Cecchin *et* *al.,* 2018) and *Chlamydomonas* *reinhardtii* where the carbon concentrating mechanism (CCM (Bogaert *et* *al.,* 2019)) and the light harvesting capacity (Perrineau *et* *al.,* 2014) is decreased by acetate along with the enhancement of respiration. While it has been reported that *G*. *sulphuraria* 074G could grow in the simultaneous presence of light and a carbon source, heterotrophy seemed to prevail in these conditions, as no photosynthetic oxygen (O_2_) production could be measured in the presence of glucose (Oesterhelt *et* *al.,* 2007).

Here, we show instead that photosynthesis and carbon metabolism (glycolysis and respiration) operate simultaneously in *Galdieria* *sulphuraria* SAG21.92 (a close relative of the 074G strain) under mixotrophic conditions, provided that the temperature conditions are kept close to the ones experienced by this alga in its natural environment. We show that performances in mixotrophy, exemplified by photosynthetic activity and biomass production, actually exceed the sum of the heterotrophic and photoautotrophic yields under limiting inorganic carbon. This synergistic effect stems from a stimulation of photosynthesis by CO_2_ of respiratory origin, which overcomes the limitation to the Calvin–Benson–Bassham cycle and suppresses photorespiration.

Limitation originates from the very low inorganic carbon concentration available in acidic conditions (pH 2) that constitute the alga’s natural growth environment. Notably, under these conditions, inorganic carbon is almost exclusively available as dissolved CO_2_ (around 10 µM) while soluble bicarbonate is virtually absent. Consistent with this hypothesis, the synergistic effect of ‘light’ and ‘dark‘ energetic metabolisms is sensitive to respiration inhibitors and is lost upon addition of exogenous CO_2_, which outcompetes endogenous CO_2_ of respiratory origin in relieving inorganic carbon limitation of the Calvin–Benson–Bassham cycle. We conclude that mixotrophy constitutes an efficient mechanism to increase intracellular CO_2_ concentration under physiological conditions, allowing *G*. *sulphuraria* to successfully exploit all the energy resources available for growth in its rather challenging environment.

## Materials and Methods

### Strains, growth and media composition

*Galdieria* *sulphuraria* SAG21.92 and 074G were obtained from the Culture Collection of Algae at Göttingen University (SAG), Germany, and were grown in sterile 2 × GS modified Allen medium, pH 2.0, containing 20 mM of sodium nitrate (NaNO_3_), and 5 mM of inorganic phosphate (K_2_HPO_4_ and KH_2_PO_4_ in a 2 : 1 ratio (Allen, 1959)) at 42°C without or with organic substrates as indicated in the text. The concentration of organic substrates was selected on the basis of data reported in the literature (Oesterhelt *et* *al.,* 2007). More precisely, glucose was employed at the concentration of 25 mM, as in Oesterhelt *et* *al*. (2007). The concentration of all the other organic compounds was adjusted to reach the same carbon atom concentration (150 mM). *Galdieria* *sulphuraria* was grown either in 250 ml flasks (50 ml culture volume), in an incubator (Infors, Bottmingen‐Basel, Switzerland, continuous light, 30 µmol photons m^−2^ s^−1^, 42°C, 100 rpm) or in a photobioreactor (Multicultivator; Photon System Instruments, Drásov, Czech Republic). Inside the multicultivator, cells were provided with air or CO_2_‐enriched air by active bubbling (see Supporting Information Methods S1). Moreover, the incident light intensity was adjusted daily to maintain constant transmitted light through the culture (see Results section). This ‘luminostat’ regime ensures maximal absorption of light without allowing a dark zone to develop inside the photobioreactor (Cuaresma *et* *al.,* 2011). Growth was monitored daily by cell counting with a LUNA cell counter (Logos Biosystems Inc., Annandale, VA, USA). Sorbitol consumption was measured using the d‐sorbitol/xylitol assay kit (Megazyme, Bray, Ireland).

### Cell fresh weight and dry weight quantification

Cell pellet was resuspended in a small volume of water and centrifuged in pre‐weighed Eppendorf tubes and the pellet was weighed. For dry weight determination fresh cell pellets were dried for three days at 60°C, weighed and expressed as g l^−1^.

### Clark electrode oxygen measurements

Oxygen exchanges in solution were measured with a Clark‐type electrode (Hansatech Instruments, King’s Lynn, UK) at 42°C. Respiration and gross photosynthesis were quantified by measuring the slope of O_2_ changes in the dark and under light exposure, respectively. Net photosynthesis was calculated assuming O_2_ consumption by the mitochondrion in the light is identical to that in the dark (Net photosynthesis = *v*_O2light_ + |*v*_O2dark_|).

### Photophysiology measurements

Photosynthetic parameters were derived from quantification of chlorophyll fluorescence emission by cultures within the multicultivator. To this aim, we employed a custom‐made fluorescence imaging system based on a previously published setup (Johnson *et* *al.,* 2009) modified as described in Methods S1. The photosynthetic electron transfer rate (ETR) (a proxy of the carbon assimilation capacity (Maxwell & Johnson, 2000)) was calculated as (Fm′ − Fs)/Fm′ × PFD, where Fm′ and Fs are the fluorescence intensities measured after exposure to a saturating pulse and in steady state, respectively, in light‐acclimated cells and PFD (photosynthetic flux density) is the incident light intensity, measured in µmol photons m^−2^ s^−1^. The cells were allowed to reach steady‐state fluorescence emissions at each intensity (5–10 min of light exposure depending on the intensity) before increasing the photon flux.

### Biochemistry and proteomic analysis

Western blot analysis was performed on cells grown for 7 d in the indicated conditions. Cells (10^9^ cells) were broken with a Precellys homogenizer (Bertin, Beaumont‐Village, France), through three cycles of 30 s at 10 000 rpm separated by a 30‐s interval. Total protein extracts were analyzed by immunoblotting with an anti‐Ribulose‐1,5‐bisphosphate carboxylase‐oxygenase (anti‐Rubisco) antibody (Agrisera, Vännäs, Sweden). An antibody against the β subunit of the ATPsynthase complex (Agrisera) was used as a loading control. 10 µg of protein was loaded per well.

For proteomic analysis, algae were cultivated under the three conditions photoautotrophy, mixotrophy and heterotrophy in parallel in the same cultivator. Three multicultivator experiments were carried out one week apart and constituted the three independent biological replicates. Cells were collected on day 7 (i.e. 4 d after addition of 25 mM d‐sorbitol to mixotrophic and heterotrophic cultures). Proteins from whole cell extracts (40 µg each) were solubilized in Laemmli buffer before being stacked in the top of a 4–12% NuPAGE gel (Life Technologies, Waltham, MA, USA), stained with R‐250 Coomassie blue (Bio‐Rad, Hercules, CA, USA) and in‐gel digested using modified trypsin (sequencing grade; Promega, Madison, WI, USA) as previously described (Bouchnak *et* *al.,* 2019). Resulting peptides were analyzed by online nanoLC‐MS/MS (Ultimate 3000 RSLCnano coupled to Q‐Exactive HF; ThermoFisher Scientific, Waltham, MA, USA) using a 200‐min gradient. Peptides and proteins were identified using mascot (v.2.6.0, Matrix Science). Spectra were searched against Uniprot (*G*. *sulphuraria* taxonomy, July 2019 version, 7347 sequences) concomitantly with a home‐made list of contaminants frequently observed in proteomics analyses (trypsins and keratins, 250 sequences).

The Proline software (Bouyssie *et* *al.,* 2020) was used to filter the results (conservation of rank 1 peptides, peptide identification false discovery rate (FDR) < 1% as calculated on peptide scores by employing the reverse database strategy, minimum peptide score of 25, and minimum of one specific peptide per identified protein group). Proline was then used to extract the MS1‐based intensities values of protein groups from unique peptides. Proteins identified in the reverse and contaminant databases (i.e. trypsin or keratin), and proteins identified with only one peptide with a score < 40 were further discarded from the list. Proteins identified in only one or two conditions were kept for analysis without statistical treatment. Proteins identified in all three conditions were submitted to statistical differential analysis using prostar (Wieczorek *et* *al.,* 2017; Wieczorek *et* *al.,* 2019). Detailed procedures are described in Methods S1.

### Metabolite extraction and analysis

Cells grown in a multicultivator were harvested at day 5 by centrifugation (4°C, 5 min and 3000 rcf), washed with ice‐cold 0.9 % (w/v) sodium chloride (NaCl), snap frozen in liquid nitrogen, and lyophilized overnight. Cells (5 × 10^8^) were disrupted in a Mixer Mill (MM 400; Retsch GmbH, Haan, Germany) for 60 s at 30 Hz using metal beads and 500 µl of ice‐cold chloroform and methanol (1 : 2.3 ratio; containing 5 µM Ribitol and 2,4‐dimethylphenylalanine (both from Sigma‐Aldrich, Munich, Germany) as internal standards) with another round of shaking in the Mixer Mill. After a 2 h incubation at −20°C, 400 µl of ice‐cold deionized water (Milli Q; Merck Chemicals GmbH, Darmstadt, Germany) were added to induce phase separation. The samples were vortexed and centrifuged for 5 min at 4°C and 16 000 rcf. The aqueous phase was transferred to a new reaction tube and the organic phase was re‐extracted with 400 µl of ice‐cold deionized water. Aqueous phases of each sample were combined and lyophilized overnight. After resuspension in 500 µl of 50% methanol 50 µl were dried in a glass inlet for analysis by gas chromatography‐mass spectrometry (GC‐MS) and ion chromatography‐mass spectrometry (IC‐MS).

For GC‐MS analysis the samples were prepared and analyzed as described by Gu *et* *al*. (2012) and Shim *et* *al*. (2020). Identification of metabolites was performed with masshunter Qualitative (v.b08.00; Agilent Technologies, Santa Clara, CA, USA) by comparing spectra to the NIST14 Mass Spectral Library (https://www.nist.gov/srd/nist‐standard‐reference‐database‐1a‐v14) and to a quality control sample containing all target compounds. Peaks were integrated using Masshunter Quantitative (v.b08.00; Agilent Technologies). For relative quantification, all metabolite peak areas were normalized to the peak area of the internal standard ribitol.

For IC‐MS a combination of a Dionex ICS‐6000 HPIC and a high field Thermo Scientific Q Exactive Plus quadrupole‐Orbitrap mass spectrometer following the method described in Schwaiger *et* *al*. (2017) and in Methods S1. Data analysis was conducted using compound discoverer (v.3.1, ThermoFisher Scientific). Setting parameters for the untargeted metabolomics workflow and peak annotation criteria are described in Methods S1.

### Labeling experiments with ^13^C‐glucose

Cells were cultivated in 250 ml Erlenmeyer flasks (50 ml culture volume) for 4 d under continuous light at 60 µmol m^−2^ s^−1^, 40°C and ambient air (0.04% CO_2_). U‐^13^C_6_‐glucose (Cambridge Isotope Laboratories Inc., Tewksbury, MA, USA) was added at day 4 in a final concentration of 25 mM and the irradiance was increased to 100 µmol m^−2^ s^−1^ either under ambient or elevated (2%) CO_2_ conditions.

Next, 1–2.5 × 10^8^ cells were harvested 1, 4, 12, 24, 36, 48 and 60 h after glucose addition as described earlier. Metabolites were extracted and measured by IC‐MS as described earlier.

Data analysis was conducted with compound discoverer (v.3.1, ThermoFisher Scientific) and the standard workflow for stable isotope labelling from compound discoverer was chosen. The default settings, which are 5 ppm mass tolerance, 30% intensity tolerance and 0.1% intensity threshold for isotope pattern matching were used and the maximum exchange rate was set to 95%.

## Results

### *Galdieria sulphuraria* metabolizes several organic substrates

Previous work (Oesterhelt *et* *al.,* 2007) has suggested that *G*. *sulphuraria* is unable to grow mixotrophically in the presence of light plus organic carbon but rather alternates between heterotrophy (in the presence of an external source of organic carbon) and phototrophy (upon inorganic carbon consumption). This conclusion was based on experiments carried out at 25°C, i.e. a temperature that is far from the physiological optimum of this alga (above 40°C), and therefore decreases photosynthetic performances (Doemel & Brock, 1971; Ford, 1979; Rossoni & Weber, 2019; Rossoni *et* *al.,* 2019b). Thus, we decided to reinvestigate the possible occurrence of mixotrophy under conditions that resemble natural growth conditions (42°C, pH 2.0) in which *G*. *sulphuraria* displays maximum photosynthetic capacity.

First, we sought compounds that could improve algal growth in presence of light. We found that several hexoses, disaccharides and pentoses, but also some polyols and amino acids (l‐alanine, l‐glutamate) were able to boost *G*. *sulphuraria*’s growth when compared to strict photoautotrophic conditions (Fig. S1). Conversely, some organic acids (malic and citric acid) and amino acids (l‐aspartate, l‐leucine, l‐valine, l‐isoleucine, l‐asparagine) had either no effect or led to a growth inhibition when compared to photoautotrophic conditions. Acetic acid was lethal to the cells. Compound concentrations were 25 mM, i.e. the same value employed in the study by Oesterhelt *et* *al.,* 2007. In the case of disaccharides, we reduced the concentration by a factor of two, to keep the overall carbon atoms concentration constant.

**Fig. 1:**
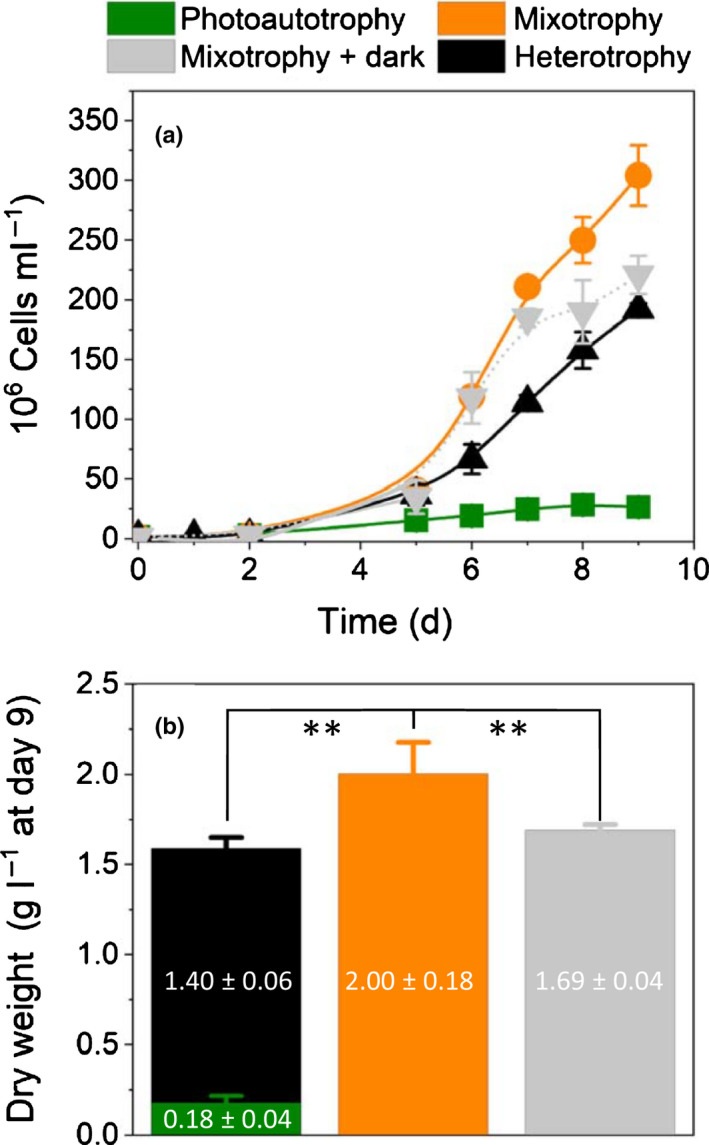
Growth enhancement of *Galdieria* *sulphuraria* SAG21.92 by reduced carbon sources is light dependent. (a) Growth curves. Data from three biological replicates ± SD. Error bars are shown when larger than the symbol size. *Galdieria* *sulphuraria* was grown in flasks at ambient CO_2_ in photoautotrophic (light only, 30 µmol photons m^−2^ s^−1^, green symbol), mixotrophic (30 µmol photons m^−2^ s^−1^ plus d‐sorbitol 25 mM, orange symbol) and heterotrophic (absence of light, presence of d‐sorbitol 25 mM, black symbol). The initial cell concentration was 1.5 × 10^6^ cells per milliliter. At day 5, the mixotrophic culture was split in two parts, and light was switched off in one culture (gray symbol). Growth was carried out at 42°C with shaking at 100 rpm, pH 2. (b) Dry weight estimated at day 9. Mixotrophic biomass (orange bar) exceeds the sum of photoautotrophic (green bar) and heterotrophic (black bar) biomass, highlighting the existence of a synergy under mixotrophic conditions. Data from three biological replicates ± SD. **Indicates that at the 0.01 level the means of the two populations (mixotrophy on one side; heterotrophy + photoautotrophy on the other one) means are statistically different (ANOVA test). The concentration of inorganic nitrogen was 20 mM, while that of inorganic phosphate was 5 mM.

Based on these results, we focused on sorbitol, a compound that boosts growth in the dark without inducing a significant loss of photosynthetic pigments (Gross & Schnarrenberger, 1995). We confirmed that sorbitol was able to sustain cell division in the dark (Fig. 1), but its effect on growth was largely enhanced by a concomitant exposure to light. The final dry weight and number of cells collected at the end of the exponential phase in the light plus carbon condition (orange symbols, Fig. 1) exceeded the sum of cells obtained in the heterotrophic (black symbols, Fig. 1) plus the photoautotrophic (green symbols, Fig. 1) conditions. This finding indicates that *G*. *sulphuraria* is not only able to perform true mixotrophy under high temperature, dim light and presence of external organic carbon sources, but that this trophic mode is highly beneficial for its growth capacity. We obtained similar effects replacing sorbitol with a monosaccharide (glucose) and a disaccharide (saccharose) (Fig. S2).

### Respiration boosts photosynthesis in mixotrophic Galdieria cells

To further characterize the consequences of mixotrophy in *G*. *sulphuraria*, we transferred half of the cells grown in mixotrophy for 5 d (Fig. 1) to heterotrophic condition and monitored growth of the four samples (photoautotrophic: green bar; heterotrophic: black bar; mixotrophic: orange bar; mixotrophic transferred to heterotrophy: gray bar) for another 5 d. While the mixotrophic cells continued to display a higher growth capacity than the heterotrophic plus photoautotrophic ones, the mixotrophic cells transferred to the dark (gray symbols) slowly reached the same cell density (Fig. 1a) and dry weight (Fig. 1b) as the heterotrophic culture, i.e. they lost the benefits provided by the simultaneous exposure to light and organic carbon within a few days. We conclude therefore that mixotrophy promotes a synergistic interaction between light and dark energy metabolisms, which slowly disappears when the light supply is halted.

**Fig. 2:**
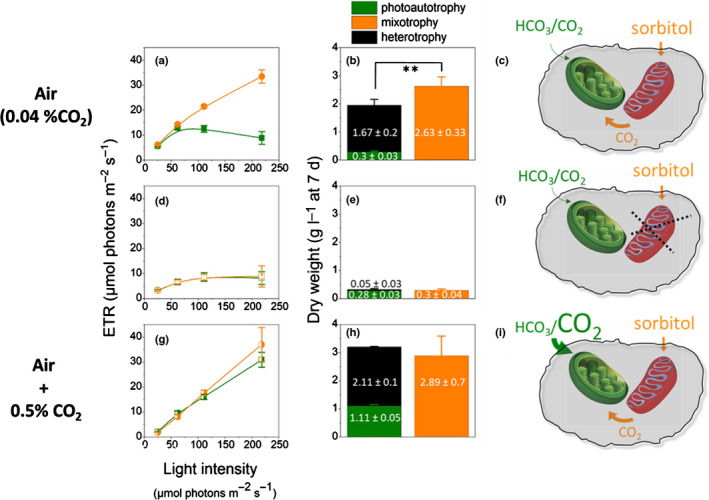
*In* *situ* measurements of photosynthetic electron transfer rate (ETR) in photoautotrophic (light) and mixotrophic (light + 25 mM d‐sorbitol) *Galdieria* *sulphuraria* SAG21.92 cells and biomass production. Cells were inoculated at 3.5 × 10^6^ cell per milliliter and grown in a photobioreactor in the light (transmitted light 10 µmol photons m^−2^ s^−1^) and air before d‐sorbitol was added in the absence (heterotrophy, black) and in the presence of light (mixotrophy, orange). Light was increased every day to keep the transmitted light to a constant value of 10 µmol photons m^−2^ s^−1^. Growth was followed at 42°C and pH 2. (a, d, g) After 5 d of growth (i.e. 2 d after the addition of d‐sorbitol), ETR was measured directly on cultures within the photobioreactor, to avoid possible temperature stress. Measurements were done in air, in the absence (a, b) and presence (d, e) of respiratory inhibitors (SHAM (1 mM) and myxothiazol (10 µM), added 24 h before measurements), or in a CO_2_‐enriched (0.5%) atmosphere (g, h). (a, d, g) Photosynthetic electron transfer: data from three biological replicates ± SD. (b, e, h) Biomass production in photoautotrophic (green, data from 12 biological replicates ± SD), heterotrophic (black, data from eight biological replicates ± SD) and mixotrophic (orange, data from eight biological replicates ± SD) conditions, respectively. Cells were collected after 7 d of growth (i.e. 4 d after addition of d‐sorbitol). (c, f, i) Sketches representing possible CO_2_ sources for photosynthesis in the three examined conditions. **Indicates that at the 0.01 level the means of the two populations (mixotrophy on one side; heterotrophy + photoautotrophy on the other one) means are statistically different (ANOVA test).

In the experiments described earlier, photoautotrophic growth was most likely limited by the low light intensity (30 µmol photons m^−2^ s^−1^), which was kept constant during growth, and therefore rapidly became limiting for photosynthesis when the cell concentration was increased in the flasks. Gas diffusion could also be limiting in flasks. Therefore, we repeated experiments in a photobioreactor (Fig. S3), where air bubbling ensured a more efficient gas delivery to the algae. Moreover, the intensity of the incident light was progressively increased in this setup to maintain a linear relationship between the cell number and the absorption. We reproduced the improvement of biomass productivity in mixotrophy with the photobioreactor (Fig. 2), where we could monitor biomass production and photosynthetic performances on actively growing cells using a custom‐built fluorescence imaging setup to monitor photosynthesis via the ETR parameter (Maxwell & Johnson, 2000) (Fig. S3).

The ETR in the photoautotrophic cultures was lower than in mixotrophic ones (Fig. 2a). The difference was larger during the first days of culturing (Fig. S4, days 4 and 5, i.e. the first and second day after sorbitol addition), and then photosynthesis progressively diminished in the mixotrophic cells (Fig. S4, days 6 and 7, i.e. the third and fourth day of mixotrophy), where we also observed a large variability in the photosynthetic capacity (Fig. S4, day 7). We ascribe this variability to a differential consumption of sorbitol, and therefore of the mixotrophic synergistic effect, in the various samples. Consistent with this hypothesis, enhancement of photosynthesis was re‐established in mixotrophic cultures, provided that sorbitol, nearly exhausted after 4 d (Fig. S5, day 7) was added again to the growth medium (Fig. S6).

To explain the synergistic effect of mixotrophy, we reasoned that photosynthesis could be limited by the availability of inorganic carbon in photoautotrophic cells. The latter is present only as CO_2_ and at very low concentration (10 µM (Gross *et* *al.,* 1998)) at ambient air in *G*. *sulphuraria*’s cultures, due to the acidic pH (pH 2). Mixotrophy could alleviate this limitation by providing extra CO_2_ of mitochondrial origin via enhanced respiration (Figs 2c, S7). We tested this hypothesis by two approaches. First, we poisoned mixotrophic and photoautotrophic cultures with myxothiazol and SHAM, known inhibitors of the cyanide sensitive and insensitive respiratory pathways, respectively, and measured consequences on photosynthetic activity. Addition of these inhibitors completely abolished the enhancement of photosynthetic activity and of biomass productivity by mixotrophy (Fig. 2d–f). Next, we increased the CO_2_ availability to the cells in mixotrophic cultures, to outcompete endogenous CO_2_ of respiratory origin with an excess of exogenous inorganic carbon. As a prerequisite for this experiment, we calibrated the CO_2_ requirement for optimum photosynthesis in our growth conditions (transmitted light of 10 µmol photons m^−2^ s^−1^). We found that photoautotrophic growth was increased by CO_2_ up to a concentration of 2% CO_2_ (Fig. S8a), the apparent affinity for CO_2_ being about 0.5%. Upon addition of external CO_2_, the photosynthetic capacity in photoautotrophic conditions increased and the biomass produced in heterotrophic plus photoautotrophic conditions became equal to that observed in mixotrophy (Fig. 2g–i). Overall, these data indicate that the synergy between respiration and photosynthesis is lost when the respiration is inhibited or when the photosynthesis becomes saturated with externally supplied CO_2_. Moreover, we hypothesize that photorespiration should be decreased to very low rates at saturating CO_2_, possibly contributing to the gain in biomass productivity at high CO_2_ concentrations. In these experiments, we also observed that the amount of biomass produced by phototrophic cells supplemented with air was the same irrespective of the light intensity employed (Fig. S8b). Conversely, biomass production could be increased by increasing the light intensity in both phototrophic cells supplemented with 0.5% CO_2_ (Fig. S8c) or in mixotrophic cells (Fig. S8d). These findings are fully consistent with the hypothesis that photosynthesis is CO_2_ limited in air, and that this limitation can be alleviated by endogenous (mixotrophy) or exogenous CO_2_.

### Metabolic acclimation to mixotrophy in G. sulphuraria

The observation that mixotrophy enhances photosynthesis to a similar level as upon addition of external CO_2_ to *G*. *sulphuraria*, suggests that mixotrophy behaves as a strategy to traffic CO_2_ from the mitochondria to the plastid, allowing this alga to successfully exploit all the energy resources available for growth and to minimize energy loss through photorespiration.

**Fig. 3:**
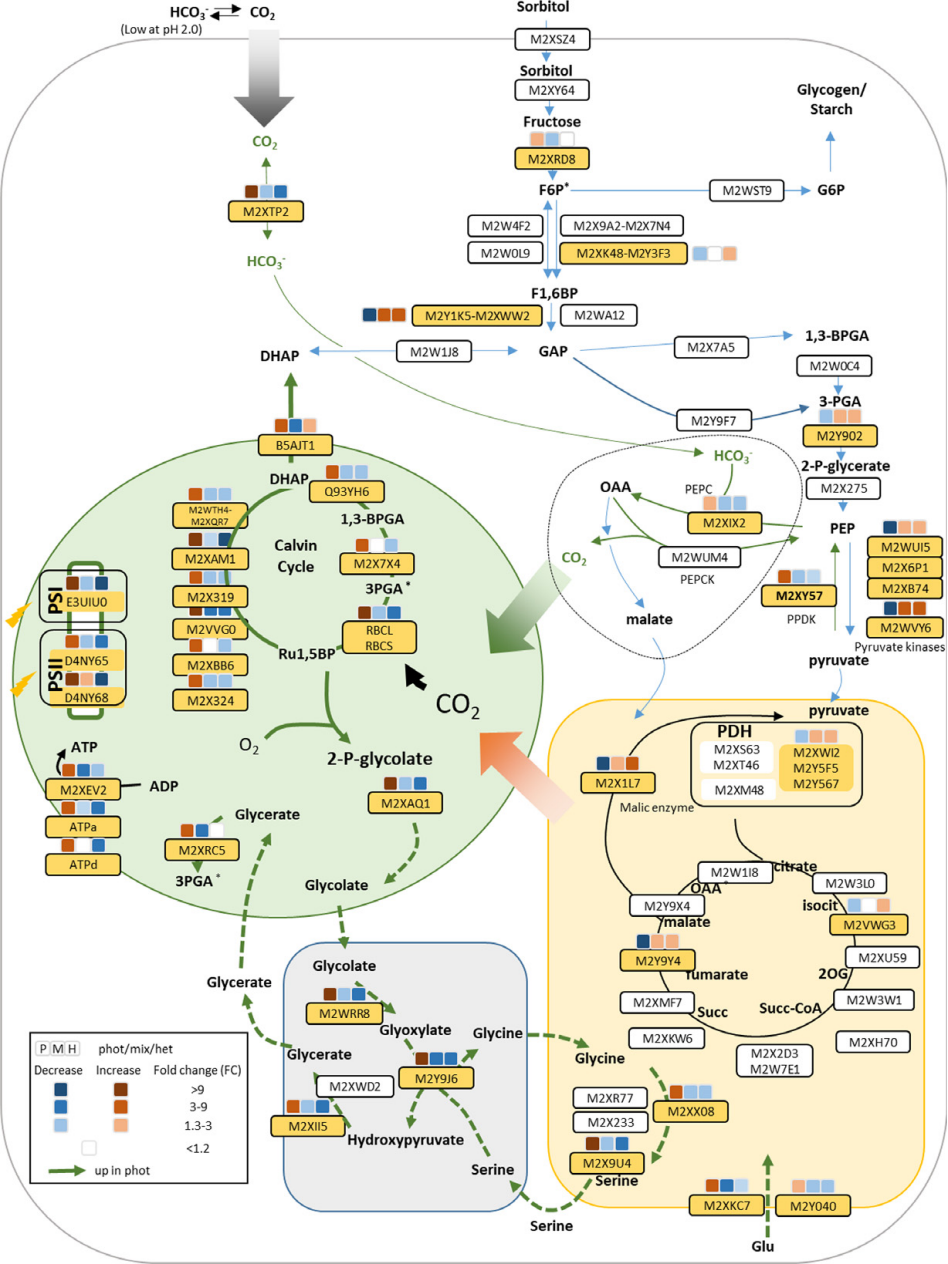
Synthesis of proteomic changes between phototrophic, mixotrophic and heterotrophic growth conditions of *Galdieria* *sulphuraria* SAG21.92. Plastid is indicated in green, cytosol in white, mitochondrion in orange and peroxisome in gray. Proteins are identified by their SwissProt accessions; boxes on top of protein names represent fold‐changes (protein average abundance in one condition was compared with the average abundance of the other two conditions – left photoautotrophy, middle mixotrophy, right heterotrophy). Proteins displaying statistically significant changes (see Methods section) are highlighted in yellow. They include proteins involved in CCM (pyruvate phosphate dikinase‐M2XY57, carbonic anhydrase‐M2XTP2, PEP carboxylase‐M2XIX2), which are much more abundant in photoautotrophic conditions than in mixotrophic or heterotrophic conditions. Enzymes involved in photorespiration – dashed arrows – (phosphoglycolate phosphatase‐M2XAQ1, glycolate oxidase‐M2WRR8, serine‐glyoxylate aminotransferase‐M2Y9J6, glycine decarboxylase P proteins‐M2X9U4, glycine/serine hydroxymethyltransferase‐M2XX08, hydroxypyruvate reductase‐M2XII5, glycerate kinase‐M2XRC5) follow the same pattern as carbon concentrating mechanism (CCM) enzymes. Conversely, pyruvate kinases (especially M2WVY6) are strongly repressed under phototrophic condition, possibly to maintain a high PEP‐oxaloacetate (OAA) pool for efficient fluxes in the carbon concentration cycle. Enzymes involved in photosynthesis are reduced under mixotrophic condition compared to photoautotrophic condition and strongly reduced under heterotrophic conditions. Mitochondrial respiratory proteins involved in the Krebs cycle or in ATP production are virtually not affected with the exception of fumarase and malic enzyme strongly reduced under phototrophic condition. Only representative proteins of the different complexes (e.g. photosynthesis, respiration) are represented. A more complete list of proteins can be found in Supporting Information Dataset S1. The complete set of proteomic data is available in Dataset S2.

To substantiate this hypothesis, we performed a complete survey of the metabolic changes between the three trophic lifestyles, and relate these changes to modifications in the cell proteome. We found that photosynthetic proteins (complexes of the electron transfer chain, enzymes of the Calvin–Benson–Bassham cycle and transporters involved in triose phosphate export) were downregulated in mixotrophic conditions compared to photoautotrophic conditions (Fig. 3), in agreement with previous suggestions (Gross & Schnarrenberger, 1995; Oesterhelt *et* *al.,* 2007). Conversely, respiratory protein levels remained relatively constant in the three conditions. This observation (photosynthetic activity is enhanced in mixotrophy despite the downregulation of the photosynthetic machinery) fully supports the conclusion that intracellular increase in CO_2_ due to enhanced respiratory activity more than compensates for the decrease in Rubisco (see also Fig. S9).

**Fig. 4:**
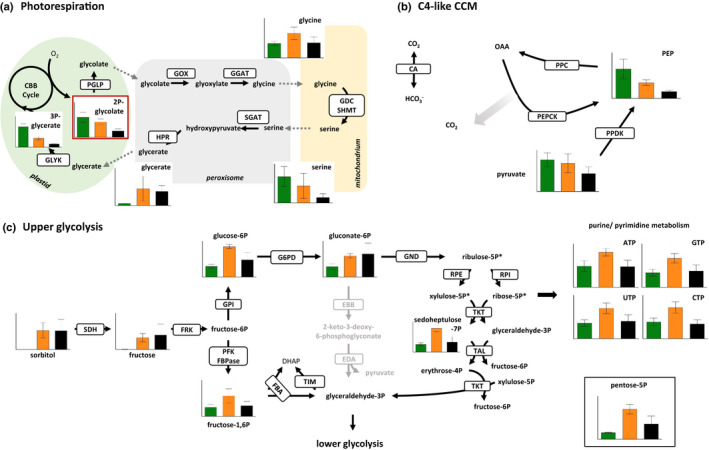
Metabolic changes between phototrophic, mixotrophic and heterotrophic growth conditions of *Galdieria* *sulphuraria* SAG21.92. Non‐phosphorylated metabolites were analyzed by gas chromatography‐mass spectrometry (GC‐MS), phosphorylated metabolites were analyzed using ion chromatography‐mass spectrometry (IC‐MS). Quantification of metabolites is provided in Supporting Information Dataset S3. Green bar: photoautotrophy; orange bar: mixotrophy; black bar: heterotrophy. (a) Changes of metabolites involved in photorespiration. 2P‐glycolate as a photorespiration‐specific metabolite is boxed in red. Glycolate, glycine, serine and glycerate are transported between cellular compartments as indicated by dashed arrows. PGLP: phosphoglycolate phosphatase; GOX: glycolate oxidase; GGAT: glutamate:glyoxylate aminotransferase; GDC: glycine decarboxylase complex; SHMT: serine hydroxymethyltransferase; SGAT: serine:glyoxylate aminotransferase; HPR: hydroxypyruvate reductase; GLYK: glycerate kinase. (b) Metabolites involved in a putative C4‐type carbon concentrating mechanism (CCM). CA: carbonic anhydrase; PPC: phosphoenolpyruvate carboxylase; PEPCK: phosphoenolpyruvate carboxykinase; PPDK: pyruvate phosphate dikinase; PEP: phosphoenolpyruvate. (c) Metabolic changes of intermediates of upper glycolytic pathways (EMP, ED, PPP) and purine/pyrimidine metabolism. The pentose‐5P (ribulose‐5P, xylulose‐5P, ribose‐5P, marked with an asterisk) could not be distinguished and are plotted in a single boxed graph. SDH: sorbitol dehydrogenase; FRK: fructokinase; GPI: glucose‐6P isomerase; G6PD: glucose‐6P dehydrogenase; GND: 6‐phosphogluconate dehydrogenase; EBB: phosphogluconate dehydratase; EDA: KDPG aldolase; PFK: 6‐phosphofructokinase; FBPase: fructose‐1,6P bisphosphatase; FBA: fructose bisphosphate aldolase; TIM: triosephosphate isomerase; RPE: ribulose‐5P epimerase; RPI: ribulose‐5P isomerase; TKT: transketolase; TAL: transaldolase. The full list of metabolite changes can be found in Dataset S3. The *Y* axes in the graphs correspond to normalized peak areas and error bars represent the standard deviation of biological quadruplicates.

In line with the hypothesis that mixotrophy channels CO_2_ from respiration to photosynthesis, proteomic analysis indicates that all the enzymes involved in photorespiration (phosphoglycolate phosphatase, glycolate oxidase, serine‐glyoxylate aminotransferase, glycine decarboxylase, glycine/serine hydroxymethyltransferase, hydroxypyruvate reductase, glycerate kinase) were less abundant in mixotrophy. This is also true for Rubisco activase (Gasu_19410; Datasets S1, S2), an enzyme that was shown to be important under low CO_2_ in *Chlamydomonas* (Pollock *et* *al.,* 2003). These findings are also corroborated by metabolite analysis (Fig. 4a). The amounts of the oxygenation product of Rubisco, 2‐phosphoglycolate are highest under photoautotrophic conditions, lower in mixotrophic conditions, and very low in heterotrophic conditions. Glycine accumulates, likely due to a higher reduction potential inside the mitochondrial matrix under mixotrophic conditions, which will reduce the rate of oxidative decarboxylation of glycine by glycine decarboxylase. Consequently, the glycine to serine ratio was inverted compared to photoautotrophic conditions. The amounts of glycerate and 3‐phosphoglycerate under mixotrophic conditions are mimicking those in heterotrophically grown cells (Fig. 4a).

Proteins involved in a putative C4‐like carbon concentrating cycle (Rademacher *et* *al.,* 2017) (Figs 3, 4b) followed the same pattern as the photorespiratory enzymes. Carbonic anhydrase and phosphoenolpyruvate carboxylase were more abundant under photoautotrophic conditions than under mixotrophic or heterotrophic conditions, and, in agreement with this finding, photoautotrophic cells also contained higher amounts of phosphoenolpyruvate (PEP) than mixotrophic or heterotrophic cells (Fig. 4b). The presence of this carbon concentrating cycle in photoautotrophic conditions could be supported by increased steady‐state levels of phosphorylated C3 compounds, as suggested by the downregulation of pyruvate kinases in phototrophy (PEP consumption might be decreased) and strong induction of pyruvate ortho‐phosphate dikinase (producing PEP). We assume that oxaloacetate (OAA) under photoautotrophic conditions is decarboxylated by PEP carboxykinase (PEPCK), and not after reduction into malate by malate dehydrogenase and decarboxylation by mitochondrial malic enzyme. This hypothesis is supported by the finding that mitochondrial malic enzyme (ME) is strongly decreased in photoautotrophy (Fig. 3) and the malate pool size is smaller under this condition than in mixotrophy or heterotrophy. We note that decarboxylation by PEPCK (as compared to ME) is energy conserving and directly yields PEP for a new round of carboxylation by PEP carboxylase.

While part of the increase in biomass production in mixotrophic conditions can be attributed to the repression of photorespiration, other benefits of the mixotrophic lifestyle may come from more carbon units being shuttled into anabolic pathways. Indeed, we observed a general increase of metabolites of the oxidative pentose phosphate pathway (ribulose‐5P, sedoheptulose‐7P) and nucleoside triphosphates (adenosine triphosphate (ATP), guanosine triphosphate (GTP), uridine triphosphate (UTP), cytidine triphosphate (CTP)) as successor metabolites (Fig. 4c). The amounts do not differ between autotrophic and heterotrophic cultures but are clearly increased under mixotrophic conditions providing evidence for a higher flux of carbon at least into purine and pyrimidine synthesis.

**Fig. 5:**
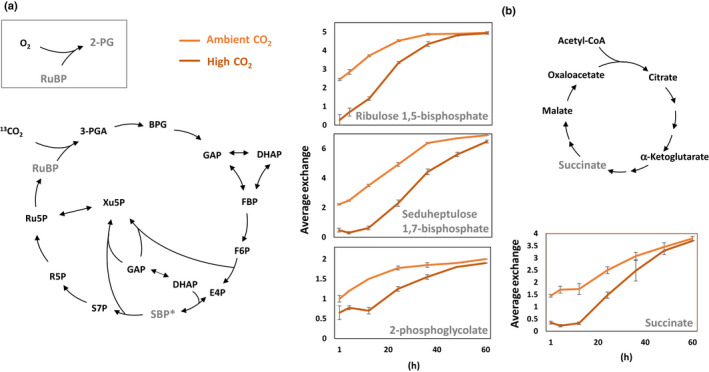
Incorporation of carbon derived from carbon‐13 (^13^C)‐labeled glucose into intermediates of the Calvin–Benson–Bassham (a) and tricarboxylic acid (b) cycles during mixotrophic cultivation of *Galdieria* *sulphuraria* SAG21.92 in two different CO_2_ concentrations. Incorporation rates are displayed as average number of labeled carbon atoms in each molecule (average exchange). Error bars represent the standard deviation of biological quadruplicates. Light orange: cells grown in ambient air (0.02% CO_2_). Dark orange cells grown in air supplied with 2% CO_2_. RuBP: ribulose 1,5‐bisphosphate; 3‐PGA: 3‐phosphoglycerate; 2‐PG: 2‐phosphoglycolate; BPG: 1,3‐bisphosphoglycerate; DHAP: dihydroxyacetone phosphate; GAP: glyceraldehyde 3‐phosphate; FBP: fructose 1,6‐bisphosphate; F6P: fructose 6‐phosphate; E4P: erythrose 4‐phosphate; Xu5P: xylulose 5‐phosphate; SBP: seduheptulose 1,7‐bisphosphate; S7P: seduheptulose 7‐phosphate; R5P: ribose 5‐phosphate; Ru5P: ribulose 5‐phosphate.

Labeling experiments using carbon‐13 (^13^C)‐labeled glucose, a sugar with similar effects on cell growth and biomass production as sorbitol (Fig. S2), further support this notion. We found a rapid incorporation of CO_2_ from ^13^C glucose into photosynthetic metabolites, such as ribulose 1,5‐bisphosphate (RuBP), sedoheptulose 1,7‐bisphosphate (SBP), and 2‐phosphoglycolate (2‐PG) (Fig. 5). While RuBP also occurs in the oxidative pentose phosphate pathway, SBP and 2‐PG are metabolites solely formed in the Calvin–Benson–Bassham cycle and can only carry a label when ^13^CO_2_ released by respiration is fixed. Incorporation of the label by shuttling of carbon backbones from the cytosol into the chloroplast is unlikely since the solute transporters in the *G*. *sulphuraria’*s chloroplast envelope do not favor the import of glycolytic intermediates under photosynthetic conditions (Linka *et* *al.,* 2008). The rate of incorporation of ^13^C is much reduced when external CO_2_ levels are increased to 2%. Interestingly, the tricarboxylic acid cycle intermediate succinate exhibits a similar pattern of delayed labeling under high CO_2_ conditions when compared to ambient air, indicating a slowed down glucose usage. Thus, the delay of label incorporation into the Calvin–Benson–Bassham cycle intermediates can be traced back to lower levels of labeled respiratory CO_2_ under CO_2_ saturated conditions.

## Discussion

At variance with a previous report (Oesterhelt *et* *al.,* 2007), *G*. *sulphuraria* cells are capable of a true mixotrophy, when transferred from strictly photoautotrophic conditions to a light and organic carbon regime, provided that the temperature conditions are kept close to the ones experienced by this alga in its natural environment. This is not only true for the *G*. *sulphuraria* SAG21.92 strain used here, but also for *G*. *sulphuraria* 074G, i.e. the strain used in the previous study by Oesterhelt *et* *al*. (2007) when tested in the presence of the same external organic carbon sources employed here (see Fig. S10). Acclimation of the strictly photoautotrophic Cyanidiophyceae *Cyanidioschyzon* *merolae* to suboptimal growth temperatures of 25°C, led to massive rearrangements of the photosynthetic apparatus and a lower photon‐to‐oxygen conversion rate when compared to cells grown at 42°C (Nikolova *et* *al.,* 2017). Also, cultivation of *G*. *sulphuraria* at 25°C led to a downregulation of transcripts encoding components of the photosynthetic machinery (Rossoni *et* *al.,* 2019b). Therefore, cultivation at suboptimal temperatures may not be in favor of high photosynthetic rates and drive Cyanidiophyceae species capable of heterotrophic growth towards this trophic state.

Mixotrophy deeply alters the carbon metabolism of *G*. *sulphuraria* cells. Under photoautotrophic conditions, CO_2_ is mainly concentrated by the PEPC/PEPCK driven CCM (Rademacher *et* *al.,* 2017). This process is repressed in mixotrophy. Moreover, enhanced respiration relieves the limitation of photosynthesis by inorganic carbon, which is at a low concentration (µmolar range) in the acid growth medium of this alga. We can quantify the extent of this process using the Clark electrode (as in Fig. S7) in a closed configuration, to avoid gas exchanges with the atmosphere (representative traces in Fig. S11a,c). In this case, the amount of O_2_ produced by photosynthesis (32.3 ± 7.5 µM and 76.6 ± 2 µM in photoautrotrophy and mixotrophy, respectively) is commensurate with the amount of CO_2_ available to Rubisco, i.e. the sum of respiratory CO_2_ (22 ± 10.5 µM and 61 ± 1.5 µM respectively, assuming a 1 : 1 stoichiometry with consumed O_2_) plus the small CO_2_ amount present in the medium (about 10 µM at pH 2) (Fig. S11c,d). These estimates clearly indicate that respiration in mixotrophy sets the rate of photosynthesis under limited CO_2_ conditions. The proximity between mitochondria and chloroplasts (highlighted in red and green, respectively, in Fig. S12) may favor this process, facilitating intracellular gas exchange between the two cell organelles, as already shown in the case of other microalgae (Lavergne, 1989).

At the same time, increased intracellular CO_2_ concentration is expected to lower the rate of photorespiration in mixotrophy. Although the Rubisco enzymes from Cyanidiophyceae show some of the highest carboxylation specificities reported to date (Sugawara *et* *al.,* 1999), photorespiration is expected to proceed at high rates at the high temperatures and low CO_2_ concentrations under which Galdieria grows. Indeed, knockout of peroxisomal glycolate oxidase in the transformable Cyanidiales alga *Cyanidioschyzon* *merolae* demonstrated that a functional photorespiratory pathway is essential for survival of these algae under ambient CO_2_ concentrations (Rademacher *et* *al.,* 2016). The observed increase in the rate of photosynthesis and gain of biomass under high CO_2_ and mixotrophic conditions (25 ± 6%, when combining data from Figs 1, 2) is consistent with overcoming the expected loss of biomass gain due to photorespiration under photoautotrophic conditions.

In conclusion, by bypassing the possible metabolic antagonism between respiration and photosynthesis, *G*. *sulphuraria* can exploit the plethora of transporters encoded by its genome (Schonknecht *et* *al.,* 2013; Rossoni *et* *al.,* 2019a) and import organic carbon available in its environment and to boost CO_2_ availability for photosynthesis. While this phenomenon certainly exists in other phototrophs (e.g. Rolland *et* *al.,* 1997), the capacity to enhance photosynthesis with respiratory CO_2_ when the latter process is increased by exogenous carbon sources could be particularly relevant in *G*. *sulphuraria*. This alga thrives in an extreme environment, where growth is limited by low pH, high temperature and possibly light availability. Dissolved inorganic carbon is very low in this hot and acidic milieu (in fact its concentration inversely correlates with the pH of different collection sites in Yellowstone National Park) (Boyd *et* *al.,* 2012; Hamilton *et* *al.,* 2012) and its light‐driven uptake is fairly low when compared to alkaline thermal habitats. Conversely, the concentration of dissolved organic carbon can be relatively high in the acidic hot springs (from 17 µM to 3 mM (Nye *et* *al.,* 2020)). Values could become much higher when this alga proliferates in mats (Gross *et* *al.,* 1998), where by‐products of every group of microorganisms may serve as ‘food’ for other groups.

Based on these considerations, it is tempting to speculate that while photosynthesis should allow cells to colonize new environments devoid of any organic carbon source, the peculiar division mode of *G*. *sulphuraria* (formation of endospores associated with the release in the media of the mother cell wall remnants), may favor mixotrophy on a longer timescale. In the acidic conditions where *G*. *sulphuraria* lives, this material is probably rapidly hydrolyzed, providing an organic carbon source to the algae, along with other external sources for dissolved organic carbon, like e.g. high‐temperature acid‐digested wood (Nye *et* *al.,* 2020). Thanks to the abundance of transporters, this alga could outcompete other microorganisms such as fungi, which are also found in these extreme conditions, for growth. Thus, the coexistence of phototrophic, mixotrophic, and heterotrophic lifestyles thanks to the subtle compromise between the activity of the two energy‐producing pathways (photosynthesis and respiration) would represent a key element for fitness and explain the success of *G*. *sulphuraria* to thrive in its extreme ecological niche. High fluctuations in the availability of dissolved organic and inorganic carbon, light, temperature, etc. in these environments possibly selected for the maintenance of a metabolic flexibility (Gross, 1999; Gross *et* *al.,* 2002; Ciniglia *et* *al.,* 2004; Cho *et* *al.,* 2020), which may have also allowed Cyanidiales to invade more moderate habitats (Yoon *et* *al.,* 2006; Azúa‐Bustos *et* *al.,* 2009; Castenholz & McDermott, 2010) and to disperse over long distances to geographically isolated extreme habitats (Rossoni *et* *al.,* 2019a).

The same metabolic flexibility opens interesting perspectives to exploit this alga for biotechnology applications. *Galdieria* *sulphuraria* has already been employed in the fields of pigment/antioxidant production, bioremediation and bioenergy (Cizkova *et* *al.,* 2019) and the possibility to exploit its lifestyle flexibility should be explored to cultivate this alga in organic matter‐rich open ponds without contamination by other microorganisms.

## Author contributions

GC, DL, APMW and GF designed research; GC, DL, EG, PW, JJ, CH, SB, DDB, JD, BG, DF, MC and GF performed research; GC, DL, PW, MT, CR, MF, APMW and GF analyzed data; GC, DL, APMW and GF wrote the article.

## Supporting information


**Dataset S1** Proteins involved in photosynthesis, central metabolism and respiration were selected from the complete proteomic dataset (see Dataset S2) and used to build Fig. 3.
**Dataset S2** Compared proteomic analysis between photoautotrophic, mixotrophic and heterotrophic growth conditions.
**Dataset S3** Compared metabolomic analysis between photoautotrophic, mixotrophic and heterotrophic growth conditions.
**Fig. S1** Consequences of different substrates on *Galdieria* *sulphuraria* growth in the light.
**Fig. S2** *Galdieria* *sulphuraria* growth in photoautotrophic, mixotrophic and heterotrophic conditions driven by a polyol, an hexose and a disaccharide.
**Fig. S3** Experimental setup to expose cells to a constant photons/cell ratio.
**Fig. S4** *In* *situ* measurements of photosynthetic electron transfer rate (ETR) in photoautotrophic (light) and mixotrophic (light + 25 mM d‐sorbitol) cells.
**Fig. S5** Enhancement of cell growth by mixotrophy and sorbitol consumption in *Galdieria* *sulphuraria*.
**Fig. S6** Mixotrophy is restored in *Galdieria* *sulphuraria* upon addition of a carbon source.
**Fig. S7** Respiration and net photosynthesis were measured every day in the three different growth conditions (photoautotrophy, mixotrophy and heterotrophy).
**Fig. S8** Biomass production as a function of transmitted light intensity and CO_2_ concentration.
**Fig. S9** Immunodetection of Rubisco in phototrophic and mixotrophic cultures under ambient and enhanced CO_2_ atmosphere.
**Fig. S10** Comparative analysis of phototrophic, mixotrophic and heterotrophic performances in *Galdieria* *sulphuraria* 074G and SAG21.92 species with d‐sorbitol and d‐glucose.
**Fig. S11** Respiration fuels photosynthesis in photoautotrophic and mixotrophic *Galdieria* *sulphuraria* cultures.
**Fig. S12** Transmission electron microscopy of *Galdieria* *sulphuraria* SAG21.92 grown 5 d under photoautotrophic, mixotrophic and heterotrophic conditions.
**Methods S1** Microalgae and media composition, growth, cell fresh weight and dry weight estimates, Clark electrode oxygen and photophysiology measurements, mass spectrometry‐based proteomic analyses, metabolic analyses by IC‐MS, electron microscopy sample preparation and observation.

 Please note: Wiley Blackwell are not responsible for the content or functionality of any Supporting Information supplied by the authors. Any queries (other than missing material) should be directed to the *New* *Phytologist* Central Office.

## Data Availability

The original contributions presented in the study are included in the article and in the Supporting Information and Supporting Information datasets. Further inquiries can be directed to the corresponding author.
