# Supplementary material for: Mixotrophic growth of the extremophile Galdieria sulphuraria reveals the flexibility of its carbon assimilation metabolism
Source: New Phytol. 2021 May 1;231(1):326–38. doi: 10.1111/nph.17359 (PMC8252106; doi:10.1111/nph.17359)
Supplement: Supplementary file 1 — Dataset S1 Proteins involved in photosynthesis, central metabolism and respiration were selected from the complete proteomic dataset (see Dataset S2) and used to build Fig. 3. Dataset S2 Compared proteomic analysis between photoautotrophic, mixotrophic and heterotrophic growth conditions. Dataset S3 Compared metabolomic analysis between photoautotrophic, mixotrophic and heterotrophic growth conditions. Fig. S1 Consequences of different substrates on Galdieria sulphuraria growth in the light. Fig. S2 Galdieria sulphuraria growth in photoautotrophic, mixotrophic and heterotrophic conditions driven by a polyol, an hexose and a disaccharide. Fig. S3 Experimental setup to expose cells to a constant photons/cell ratio. Fig. S4 In situ measurements of photosynthetic electron transfer rate (ETR) in photoautotrophic (light) and mixotrophic (light + 25 mM d‐sorbitol) cells. Fig. S5 Enhancement of cell growth by mixotrophy and sorbitol consumption in Galdieria sulphuraria. Fig. S6 Mixotrophy is restored in Galdieria sulphuraria upon addition of a carbon source. Fig. S7 Respiration and net photosynthesis were measured every day in the three different growth conditions (photoautotrophy, mixotrophy and heterotrophy). Fig. S8 Biomass production as a function of transmitted light intensity and CO2 concentration. Fig. S9 Immunodetection of Rubisco in phototrophic and mixotrophic cultures under ambient and enhanced CO2 atmosphere. Fig. S10 Comparative analysis of phototrophic, mixotrophic and heterotrophic performances in Galdieria sulphuraria 074G and SAG21.92 species with d‐sorbitol and d‐glucose. Fig. S11 Respiration fuels photosynthesis in photoautotrophic and mixotrophic Galdieria sulphuraria cultures. Fig. S12 Transmission electron microscopy of Galdieria sulphuraria SAG21.92 grown 5 d under photoautotrophic, mixotrophic and heterotrophic conditions. Methods S1 Microalgae and media composition, growth, cell fresh weight and dry weight estimates, Clark electrode oxygen and photo [file NPH-231-326-s003.pdf]

Supporting Information for

MIXOTROPHIC GROWTH OF THE EXTREMOPHILE *GALDIERIA SULPHURARIA* REVEALS THE FLEXIBILITY OF ITS CARBON ASSIMILATION METABOLISM

Gilles Curien[1]*, Dagmar Lyska[2], Erika Guglielmino[1], Phillip Westhoff[2], Janina Janetzko[2], Marianne Tardif[3], Clément Hallopeau[1], Sabine Brugière[3], Davide Dal Bo[1], Johan Decelle[1], Benoit Gallet[4], Denis Falconet[1], Michele Carone[5], Claire Remacle[5], Myriam Ferro[3], Andreas P.M. Weber[2], Giovanni Finazzi[1]

[1]Laboratoire de Physiologie Cellulaire et Végétale. Univ. Grenoble Alpes, CNRS, CEA, INRA, 38054 Grenoble Cedex 9, France

[2]Institute of Plant Biochemistry, Cluster of Excellence on Plant Sciences (CEPLAS), Heinrich Heine University, Düsseldorf, Germany

[3]EdyP Laboratoire Biologie à Grande Echelle. Univ. Grenoble Alpes, CEA, Inserm, BGE U1038, 38054 Grenoble Cedex 9, France

[4]Institut de Biologie Structurale, Univ. Grenoble Alpes, CNRS, CEA, 71 Avenue des Martyrs, 38044 Grenoble, France

[5]Genetics and Physiology of microalgae, InBios/Phytosystems Research Unit, University of Liege, Belgium

* Corresponding author: Gilles Curien. phone: +33 4 38782509, email: gilles.curien@cea.fr

article acceptance date: 18 March 2021.

This file includes:

Methods S1
Figures S1 to S12
Legends for Datasets S1 to S3

Other supplementary materials for this manuscript include the following:

Datasets S1 to S3

Methods S1

Microalgae and media composition

Galdieria sulphuraria SAG21.92 and 074G were obtained from the Culture Collection of Algae at Göttingen University (SAG) and was grown in sterile 2xGS modified Allen medium, pH 2.0, containing 20 mM of $NaNO_3$, and 5 mM of inorganic phosphate (K_2HPO_4 and KH_2PO_4 in a 2/1 ratio, (Allen, 1959)) at 42°C. The medium and flasks were sterilized in an autoclave for 20 min at 121 °C in order to prevent any contamination. For the experiments with different organic carbon sources, the 2xGS modified Allen media was supplemented by adding filter-sterilized concentrated organic substrate solutions as indicated in the text. The concentration of organic substrates was selected on the basis of data reported in literature (Oesterhelt *et al.*, 2007). *G. sulphuraria* was grown either in 250 mL flasks (50 mL culture volume) in an incubator (Infors, Switzerland, continuous light, 30 µmol photons m^{-2} s^{-1}, 42°C, 100 rpm) or in a multicultivator (Photon System Instruments, Czech Republic). For growth in the multicultivator, cells were provided with air or CO_2-enriched air (see below) bubbled into the solution ensuring both aeration and mixing. Culture volume was 80 mL. Incident light intensity was adjusted daily to maintain constant transmitted light thus offering a constant light intensity to the cells (see Results and below). The fraction of CO_2 in the feed air was regulated by a flow metering system (Photon System Instruments, Czech Republic). The total gas flow was 0.4 L/min for each culture.

Growth

Cultures used to inoculate flasks or multicultivator tubes were grown photoautotrophically in the air in flasks placed in the incubator. Adequate volumes of cultures at a density of 7 x 10^7 cells per mL were centrifuged and resuspended in a fresh medium to obtain an initial cell concentration of 1.5 x 10^6 cells per mL (in flasks) or 3.5 x 10^6 cells per mL (multicultivator). For organic substrates screening in flasks under mixotrophic conditions (see Fig. S1 & S2), substrate was added at a concentration of 150 mM carbon atom (*e.g*. 12.5 mM saccharose). For growth in the multicultivator cells were adapted for 3 days with an incident light of 60 µmol

photons m^{-2} s^{-1} (transmitted light ~ 10 µmol photons. $m^2.s^{-1}$), before addition of organic carbon (150 mM C) for the mixotrophic and heterotrophic conditions. For the heterotrophic condition, light was switched-off and tubes were covered with aluminum foil to keep them in the dark. For experiments with the multicultivator, the incident light intensity was adjusted daily to maintain constant transmitted light though the culture (see Results). This 'luminostat' regime should ensure maximal absorption of light without allowing a dark zone to develop inside the multicultivator (Cuaresma *et al.*, 2011) (see Supplementary Fig. S3). For most conditions, a transmitted light of 10 µmol photons m^{-2} s^{-1} was used. Tests were carried out in the range from 5 to 30 µmol photons m^{-2} s^{-1} (see Supplementary Fig. S8). Experiments were either carried out in air or in air enriched with CO_2 (0.5 to 4%, see Supplementary Fig. S8). Sorbitol consumption was measured using the D Sorbitol/Xylitol assay kit (Megazyme).

To monitor algal growth, samples were taken daily and growth was estimated using a LUNA™ cell counter (Logos Biosystems, Inc. USA).

Cell fresh weight and dry weight estimates

Cells were collected by centrifugation in 50 mL tubes, cell pellet was resuspended in a small volume of water and centrifuged in pre-weighted eppendorf tubes and pellet was weighted. For dry weight determination fresh cells pellets were exposed for three days at 60°C, weighted and expressed as g L^{-1}.

Clark electrode oxygen measurements

Net oxygen exchanges in solution were measured with a Clark-type electrode (Hansatech Instruments, UK) at 42°C. The electrode equilibrated for 12 h in distilled water was calibrated at 42°C in the air (100% of O_2) and by bubbling argon (0% of O_2). For oxygen exchanges measurements, an aliquot of the cell culture was collected immediately before the measurement (30 10^6 cells) and centrifuged at 42°C. The pellet was resuspended in 1 mL 2 x GS medium, pH 2.0, 42°C and introduced in the measure chamber thermostated at 42°C. The electrode was closed and measurement was first carried out in the dark for 10 minutes before light was switched on (320 µmol photons m^{-2} s^{-1}). Respiration and gross

photosynthesis were quantified by measuring the slope of oxygen changes in the dark and under light exposure. Net photosynthesis was calculated assuming O_2 consumption by the mitochondrion in the light is identical to that in the dark (Net photosynthesis = $v_{O2light}$ +|v_{O2dark}|).

Photophysiology measurements

Photosynthetic parameters were derived from quantification of chlorophyll fluorescence emission by cultures within the multicultivator. To this aim, we employed a custom-made fluorescence imaging system based on a previously published setup (Johnson *et al.*, 2009). The system was modified by replacing the green LEDs providing actinic light with orange LEDs (emission peak 630 nm, Full Width at Half Maximum: 40 nm), and the acquisition setup with a GigE µeye camera (IDS, Germany). Measuring light was provided by a LED peaking at 590 nm, to maximize excitation of the phycobiliprotein complexes of Galdieria. The photosynthetic electron transfer rate, ETR_{PSII}, was calculated as the product of the light intensity times the photochemical yield in the light (Fm'-Fs)/Fm' x PFD, where Fm' and Fs are the fluorescence intensities measured after exposure to a saturating pulse and in steady state, respectively, in light-acclimated cells and PFD (Photosynthetic Flux Density) is the incident light intensity, measured in µmol photons m-2 s-1.(see (Maxwell & Johnson, 2000) for more details). Cells were allowed to reach steady state fluorescence emissions at every light (5-10 minutes of light exposure depending on the intensity) before increasing the photon flux.

Mass spectrometry-based proteomic analyses

<u>Experimental design</u>

Galdieria sulphuraria SAG21.92 was cultivated under the three conditions (photoautotrophy, mixotrophy and heterotrophy) in parallel in the same cultivator (2-3 tubes per condition). Three multicultivator experiments were carried out one week apart and constituted the biological replicates. Only one tube per condition and per cultivator was chosen having the closest ODs within the same condition.

Cell breakage was performed on 10^9 cells with a Precellys homogenizer (Bertin, France).

Protein digestion

Each protein sample (40 µg) was stacked by a 1 cm-migration on the top of a NuPAGE 4–12% gel, (Invitrogen) before Coomassie blue staining (R250, Bio-Rad). Gel bands of concentrated proteins were manually excised and cut into pieces before being washed by 6 successive incubations of 15 min in 25 mM NH_4HCO_3 containing 50% (v/v) acetonitrile. Gel pieces were then dehydrated in 100% acetonitrile and incubated at 53 °C with 10 mM DTT in 25 mM NH_4HCO_3 for 45 min and in the dark with 55 mM iodoacetamide in 25 mM NH_4HCO_3 for 35 min. Alkylation was stopped by adding 10 mM DTT in 25 mM NH_4HCO_3 and mixing for 10 min. Gel pieces were then washed again by incubation in 25 mM NH_4HCO_3 before dehydration with 100% acetonitrile. Modified trypsin (Promega, sequencing grade) in 25 mM NH_4HCO_3 was added to the dehydrated gel pieces for an overnight incubation at 37 °C. Peptides were then extracted from gel pieces in three 15-min sequential extraction steps in 30 µl of 50% acetonitrile, 30 µl of 5% formic acid and finally 30 µl of 100% acetonitrile. The pooled supernatants were then vacuum-dried.

MS/MS analysis

The dried extracted peptides were resuspended in acetonitrile 5%, trifluoroacetic acid 0.1% and analyzed via online nano-LC-MS/MS (nano-liquid chromatography-tandem mass spectrometry; Ultimate 3000 RSLCnano and Q-Ex HF, Thermo Fischer Scientific, Thermo Scientific, Waltham, MA, USA). Peptide mixtures were desalted on line using a reverse phase precolumn (PepMap C18 Thermo Fisher Scientific) and resolved on a C18 column (ReproSil-Pur 120 C18-AQ 1.9 µm column, Dr. Maisch GmbH). The nanoLC method consisted in a 200-min gradient at a flow rate of 300 nL·min−1 ranging from 5.1% to 72.2% acetonitrile in 0.08% formic acid in 182 min. This gradient was operated as multi-steps optimized by GOAT software (V1.0.1) for Galdieria whole cell extracts. MS (mass spectrometry) and MS/MS data were acquired using the Xcalibur software (Thermo Fisher

Scientific). The spray voltage was set at 2 kV and the heated capillary was adjusted to 270 °C. Survey full-scan MS spectra (m/z = 400–1600) were obtained in the Orbitrap with a resolution of 60,000 after accumulation of 10^6 ions (maximum filling time: 200 ms). The 20 most intense ions from the preview survey scan delivered by the Orbitrap were fragmented via collision-induced dissociation in the LTQ after accumulation of 1e5 ions (maximum filling time: 50 ms).

Database searches and quantification

Data were processed automatically using the Mascot Distiller software (version 2.7.1.0, Matrix Science). Peptides and proteins were identified using Mascot (version 2.6.0) through concomitant searches against Uniprot (*Galdieria sulphuraria* taxonomy, July 2019 version), classical contaminants database (homemade) and their corresponding reversed databases. Trypsin/P was chosen as the enzyme and three missed cleavages were allowed. Precursor and fragment mass error tolerance were set, respectively, to 10 ppm and 25 mmu. Peptide modifications allowed during the search were: carbamidomethylation (fixed), acetyl (protein N-terminal, variable) and methionine oxidation (variable). The Proline software ((Bouyssie *et al.*, 2020), http://proline.profiproteomics.fr) was used to filter the merged results: conservation of rank 1 peptide-spectrum match (PSM) with a minimal length of 7 and a minimal score of 25. PSM score filtering is then optimized to reach a False Discovery Rate (FDR) of PSM identification below 1% by employing the target decoy approach. A minimum of one specific peptide per identified protein group was set. Proline was then used to perform MS1-based label free quantification of the peptides and protein groups. The "Cross-assignment" option was activated and allowed within replicates of the same condition only. Intensity values were extracted from specific peptides and used to infer protein abundances from the different samples. An ultimate filter was applied to discard non-relevant or doubtful identifications. This concerns proteins identified in the reverse and contaminant databases, proteins identified with only one peptide with a Mascot score inferior to 40 and few unquantified proteins. Proteins identified in the reverse and contaminant databases (i.e. trypsin or keratin), and proteins identified with only 1 peptide with a score <40 were further discarded from the list.

Statistical treatment

The remaining list was considered for bioanalysis. Since there were many missing proteins in the photoautotrophy condition compared to the other two conditions, the list was divided into two sets. One set on which no statistical treatment was applied was constituted of proteins entirely missing in one condition (having no abundance values in all 3 replicates). The other set (proteins having at least 1 abundance value in all three conditions) was used for differential analysis using ProStaR (Wieczorek *et al.*, 2017; Wieczorek *et al.*, 2019). For this, the intensity values were log2 transformed and normalized by Variance Stabilizing Normalization (VSN) algorithm (Huber *et al.*, 2002) with the « overall » option. The missing abundances were imputated according to the Structured Least Square Adaptative (SLSA) method. The Limma test was applied for comparison of one condition versus the other two combined. Due to huge differences in the matrix – proteins identified and intensities – in photoautotrophy compared to mixotrophy or heterotrophy, the strategy consisting of comparing one condition to the average of the other two (option "One_vs_All" in ProStaR) gave better discriminating results than comparisons of one condition to another one condition ("One_vs_One" option in ProStaR). Differentially recovered proteins were sorted out using a log2 fold-change (FC) cut-off of 1 and a p-value threshold (on the remaining proteins) that guarantees a Benjamini–Hochberg FDR of approximately 0.01%. The variations in abundance between the three cultivation conditions are important which made it difficult to objectively determine the latter threshold. We deliberately chose a very low FDR threshold to select a protein set of reasonable size in each comparison.

Metabolic analyses by IC-MS

The dried sample was reconstituted in 100 µL deionized water and 5 µL were injected via a Dionex AS-AP autosampler in push partial mode with a 10 µL loop. The temperature of the autosampler was set to 4 °C to maintain sample stability. Anion exchange chromatography was conducted on a Dionex IonPac AS11-HC column (2 mm × 250 mm, 4 µm particle size, Thermo Scientific) equipped with a Dionex IonPac AG11-HC guard column (2 mm × 50 mm, 4 µm, Thermo Scientific)

at 30 °C. The mobile phase was established using an eluent generator with a potassium hydroxide cartridge to produce a potassium hydroxide gradient. The column flow rate was set to 380 µL min^{-1} with a starting KOH concentration of 10 mM. The concentration was held for 3 min, then increased to 50% within 9 min followed by a steeper increase to 100% within 7 min. After 2 min of plateau the concentration dropped immediately back to 10 % for 8 min of equilibration. To prevent the high amounts of salt from entering the ESI source a Dionex ADRS 600, 2 mm suppressor was used in dynamic mode at a temperature of 15 °C. Spray stability was achieved with a make up consisting of methanol with 10 mM acetic acid delivered with 150 µL min^{-1} by an AXP Pump. The electro spray was achieved in the ESI source using the following parameters: sheath gas 30, auxiliary gas 15, sweep gas 0, spray voltage - 2.8 kV, capillary temperature 300 °C, S-Lens RF level 45, and auxiliary gas heater 380 °C. For the untargeted approach the mass spectrometer operated in a combination of full mass scan and a data-dependent Top5 MS2 (ddMS2) experiment. The full scan (60-800 m/z) was conducted with a resolution of 140.000 and an automatic gain control (AGC) target of 10^6 ions with a maximum injection time (IT) of 500 ms. The Top5 ddMS2 experiment was carried out with a resolution of 17.500 and an AGC target of 10^5 and a maximum IT of 50 ms. The stepped collision energy was used with the steps (15,25,35) to create an average of NCE 25.

Untargeted data analysis was conducted using Compound Discoverer (version 3.1, Thermo Scientific) using the "untargeted Metabolomics workflow". In this workflow automatic retention time alignment is performed in a window of 2 min within 5 ppm mass accuracy as well as unknown compound detection, and compound grouping across all samples. Elemental compositions are predicted based on accurate mass and chemical background is substracted by using blank extraction samples. Levels of peak annotation are indicated in the supplementary Dataset S3. Lowest level of annotation is based on accurate mass (3 ppm mass accuracy) on MS^1 level and allows to evaluate potential sum compositions and comparisons with databases such as Chemspider (http://www.chemspider.com) with ranking by the mzLogic algorithm. The next level of identification is via MS^2

level by comparing MS^2 fragment spectra using mzCloud (ddMS2) (match factor 50) and MS^1 level ChemSpider (by formula or exact mass). Highest identification level via an in-house MS^2 spectral library and retention time was established using mzVault. QC-based batch normalization was performed with QC sample injection every 5 samples during the sequence. Differential analysis, determination of p-values, adjusted p-values, ratios, fold change, were also calculated using Compound Discoverer.

Labelling experiments with ^{13}C-glucose

Cells were cultivated in 250 mL Erlenmeyer flasks (50 mL culture volume) for four days under continuous light at 60 µmol m-2 s-1, 40 °C and ambient air (0.04% CO_2). U-$^{13}C_6$-glucose (Cambridge Isotope Laboratories Inc, Tewksbury, Massachusetts) was added at day 4 in a final concentration of 25 mM and the irradiance was increased to 100 µmol m-2 s-1 either under ambient or elevated (2%) CO_2 conditions.

1-2.5 x108 cells were harvested 1, 4, 12, 24, 36, 48 and 60 hours after glucose addition as described above. Metabolites were extracted and measured by IC-MS as described above.

Data analysis was conducted with Compound Discoverer (version 3.1, Thermo Scientific) and the standard workflow for stable isotope labelling from Compound Discoverer was chosen. The default settings, which are 5 ppm mass tolerance, 30 % intensity tolerance and 0.1 % intensity threshold for isotope pattern matching were used and the maximum exchange rate was set to 95%.

Electron microscopy sample preparation and observation

Cells were pelleted (1000g, 5 min, 4°C) and fixed in reduced osmium tetroxide. To this end, cell pellets were resuspended in 0.1 M phosphate buffer (pH 7.4) and 2.5% glutaraldehyde and incubated overnight at 4°C. Cells were then pelleted and washed five times in 0.1 M phosphate buffer. Cells were fixed by a 1h incubation on ice in 500 µL 0.1 M phosphate buffer containing 1% osmium and 1.5% ferricyanide potassium red before they were pelleted and washed five times with

0.1 M phosphate buffer. Pellets were resuspended in 0.1 M phosphate buffer containing 0.1% tannic acid and incubated for 30 min in the dark at room temperature. Again, cells were pelleted and washed five times with 0.1 M phosphate buffer. The samples were dehydrated in ascending sequences of ethanol and infiltrated with ethanol/Epon resin mixture. Finally, the cells were embedded in Epon. Ultrathin sections (70 nm) were prepared with a diamond knife on a PowerTome ultramicrotome (RMC products) and collected on nickel grids. Ultrathin sections were examined on a Philips CM120 transmission electron microscope operating at 80 kV.

Figures S1 to S12

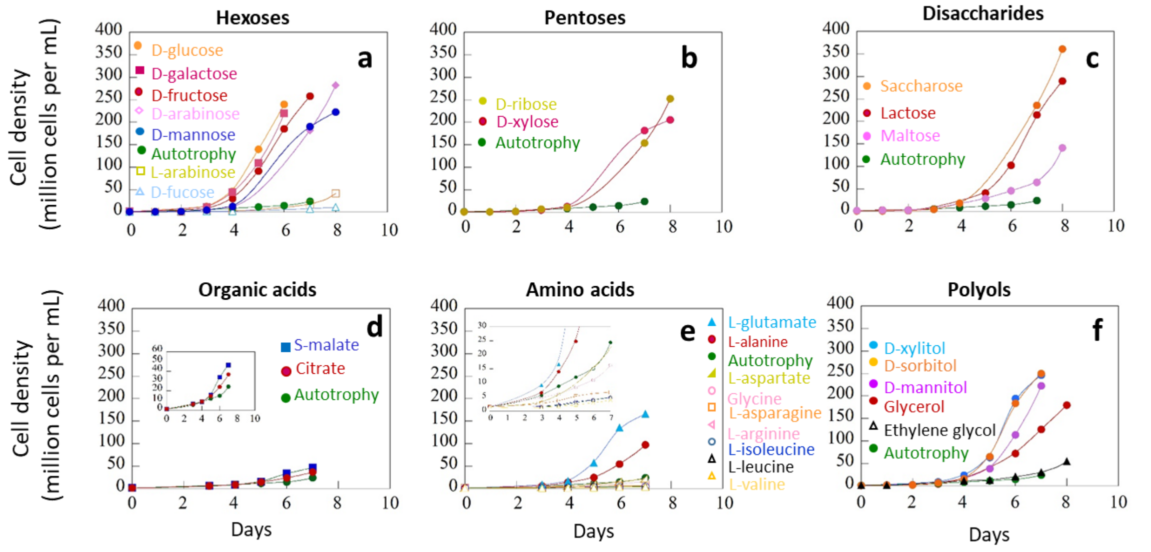

Figure S1: Consequences of different substrates on *G. sulphuraria* SAG21.92 growth in the light. Growth was measured in flasks at ambient CO_2 in the presence of 25 mM reduced carbon source added from the beginning of the culture (day 0). Cultures were inoculated at a cell density of 1.5 10^6 photoautotrophic cells mL^{-1}. Growth of photoautotrophic cells is indicated in green, for comparison. Light intensity was 30 µmol photons m^{-2} s^{-1}. Experiments were carried out at 42°C with shaking at 100 rpm, pH 2. **a**, hexoses, **b**, pentoses, **c**, disaccharides, **d**, organic acids, **e**, amino acids, **f**, polyols. Inserts in panel **d** and **e** are zooms on the data.

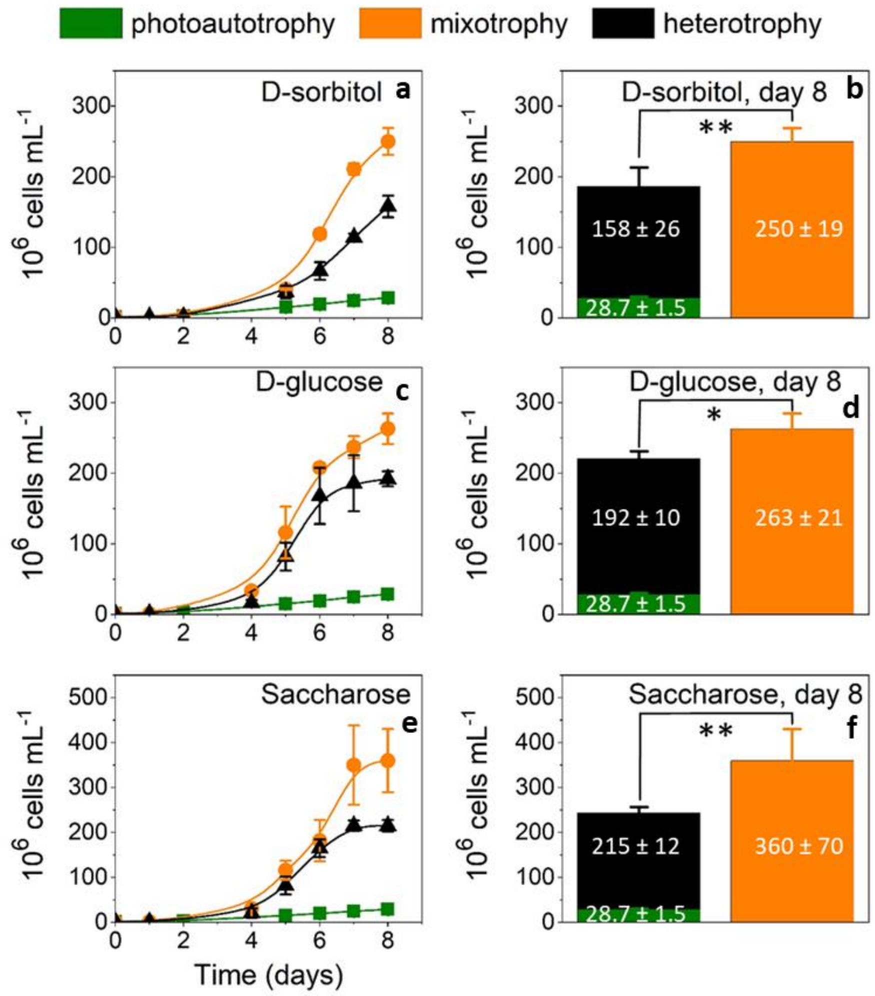

Figure S2: *G. sulphuraria* SAG21.92 growth in photoautotrophic, mixotrophic and heterotrophic conditions driven by a polyol, an hexose and a disaccharide. Cells were grown in flasks, in photoautotrophic (light only, 30 µmol photons m^{-2} s^{-1}, green), mixotrophic (30 µmol photons. $m^{-2}.s^{-1}$ plus a source of reduced carbon, orange) and heterotrophic (absence of light, presence of a source of reduced carbon, black) conditions at ambient CO_2, 42°C and pH 2. **a,b**: D-sorbitol 25 mM; **c,d**: D-glucose 25 mM; **e,f**: saccharose 12.5 mM. **b-d-f**: Mixotrophic growth (orange bars) exceeds the sum of photoautotrophic (green bars) and heterotrophic (black bars) growth, highlighting the existence of a synergy under mixotrophic conditions. Cultures were started from photoautotrophic cells, which were diluted at 1.5 10^6 cells mL^{-1} for each condition. Experiments were carried out at 42°C with shaking at 100 rpm. **b, d, f**, Final cell concentration (day 8) in photoautotrophic, heterotrophic and mixotrophic conditions. Data from 3 biological replicates ± S.D. * indicate that at the 0.05 level the means of the two populations (mixotrophy on one side, heterotrophy+photoautotrophy on the other one) means are statistically different (Anova test). ** indicate that at the 0.01 level the means of the two populations (mixotrophy on one side, heterotrophy+photoautotrophy on the other one) means are statistically different (Anova test).

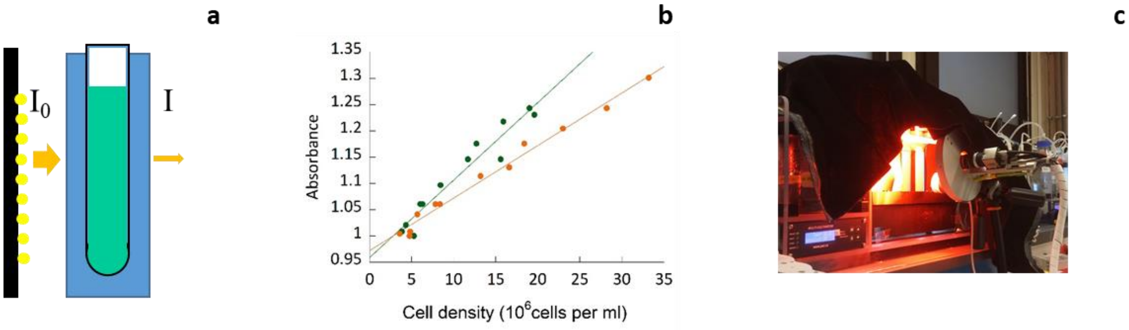

Figure S3: Experimental setup to expose cells to a constant photons/cell ratio. **a**, Schematic side view of the photobioreactor with the LED panel (yellow spots), water bath (blue) and culture tube (green). I_0 and I represent the incident light (settings on the device) and the light transmitted through the device, respectively. I_0 was progressively increased during cell growth, to keep a linear relation between cell density and absorption. **b**, Relation between absorbance and cell number under mixotrophic and photoautotrophic conditions for I = 10 µmol photons m^{-2} s^{-1}. The slopes differ for mixotrophic (orange) and photoautotrophic (dark green) cells possibly because the photosynthetic apparatus is down-regulated in the presence of organic carbon. The lower amount of photosynthetic complexes reduces light absorption for the same amount of cell. **c**, Custom-made setup to evaluate photosynthetic performances of *G. sulphuraria* cells within the photobioreactor. The system is equipped with orange LEDs (λ = 590 nm), to enhance absorption by the phycobiliprotein complexes of *G. sulphuraria*, and a near infrared camera to measure fluorescence emitted by the algae inside the photobioreactor (PBR tubes).

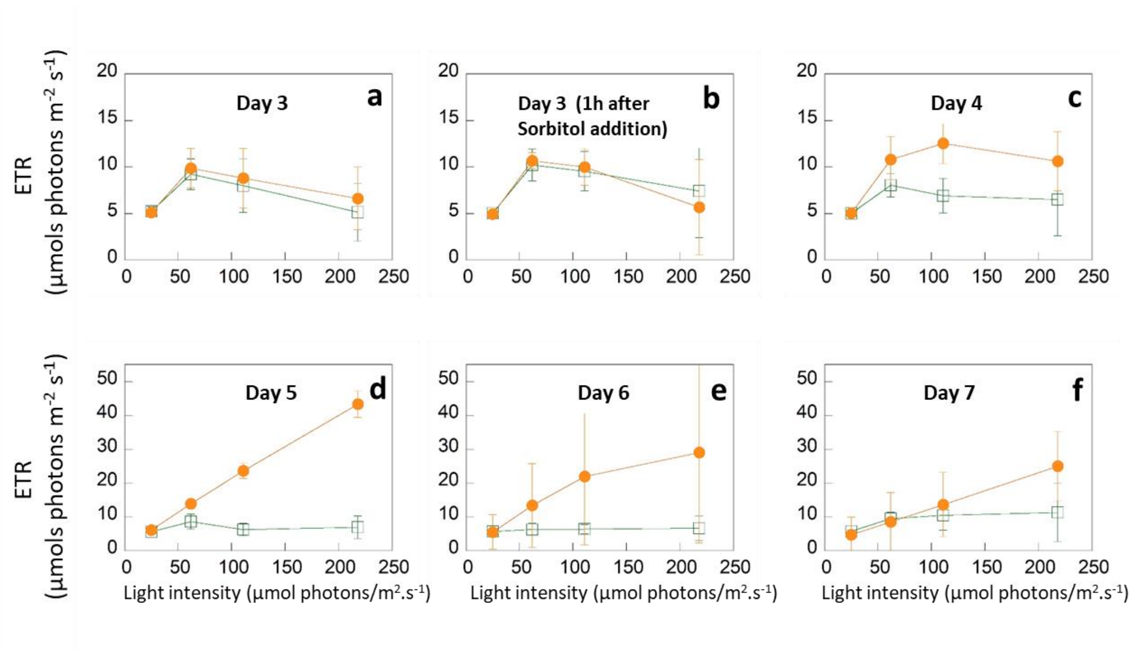

Figure S4: *In situ* measurements of photosynthetic electron transfer rate (ETR) in photoautotrophic (light) and mixotrophic (light + 25 mM D-sorbitol) *G. sulphuraria* SAG21.92 cells. Cells were inoculated at 3.5 x 10^6 cell mL^{-1} and grown in the light (transmitted light 10 µmol photons m^{-2} s^{-1}) and air for three days before D-sorbitol was added (mixotrophy, orange) or not (autotrophy, green). Light was increased every day to keep the transmitted light to a constant value of 10 µmol photons m^{-2} s^{-1}. **a-f**, After three days of growth, ETR was followed every day directly on cultures within the photobioreactor, to avoid possible temperature stress. Data from 3 biological replicates ± S.D.

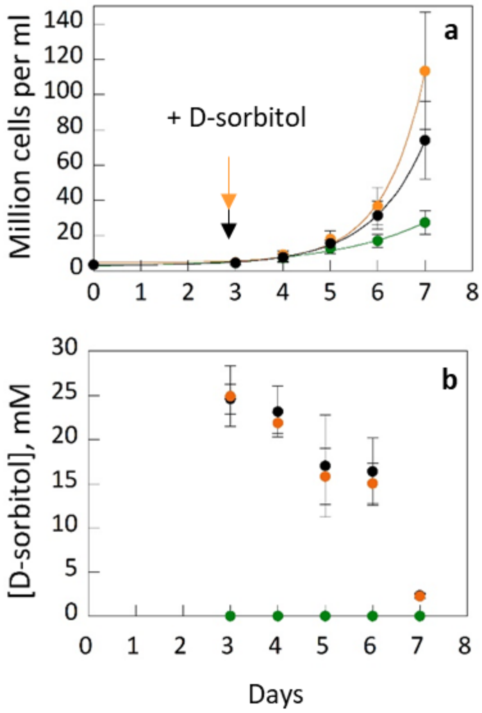

Figure S5: Enhancement of cell growth by mixotrophy and sorbitol consumption in *G. sulphuraria* SAG21.92. *G. sulphuraria* was inoculated at 3.5 10^6 cells mL^{-1}. At day 3, cultures were either continued without any addition (photoautotrophy, green), or supplemented with 25 mM D-sorbitol in the presence of light (mixotrophy, orange) or in the dark (heterotrophy, black). **a,** Growth curves (data from 12 biological replicates ± S.D.). Growth rates were 0.5 ± 0.1 day^{-1}; 1.2 ± 0.1 day^{-1} and 0.9 ±0.05 day^{-1} in photoautotrophic, mixotrophic and heterotrophic cultures, respectively. **b**, Sorbitol consumption in mixotrophic and heterotrophic cultures. Data from 3 biological replicates ± S.D.

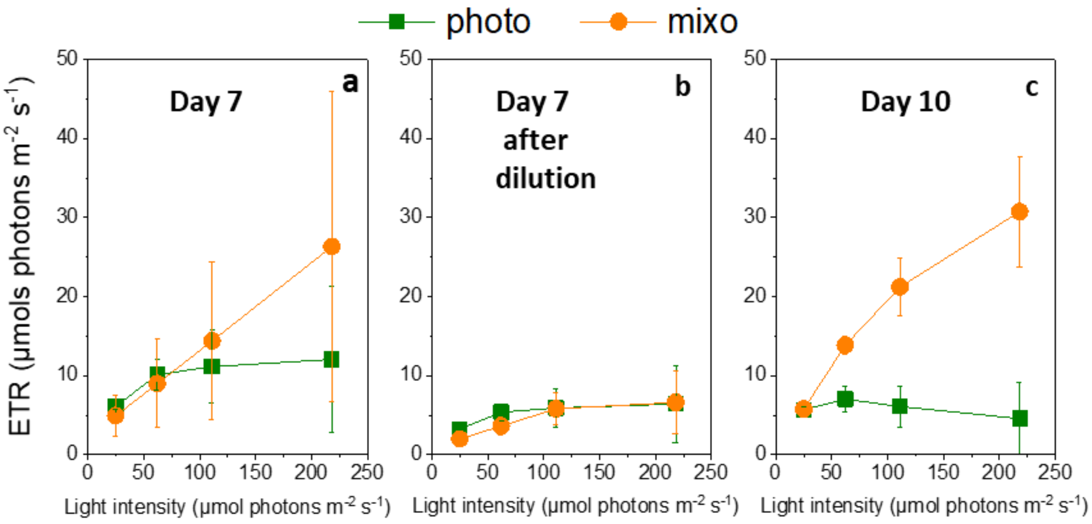

Figure S6: Mixotrophy is restored in *G. sulphuraria* SAG21.92 upon addition of a carbon source. a, Mixotrophic cells (orange) were let consuming sorbitol (day 7, *i.e*. 4 days after addition of D-sorbitol). **b,** Cells at day 7 were diluted to 3.5 x 10^6 cells mL^{-1}, and growth was resumed in the light (transmitted light 10 µmol photons m^{-2} s^{-1}) without D-sorbitol (photoautotrophy, green) or in the presence of 25 mM D-sorbitol (orange). ETR measurements carried three days later **(c)** indicate that mixotrophic photosynthetic capacity was fully restored. Data from 3 biological replicates ± S.D.

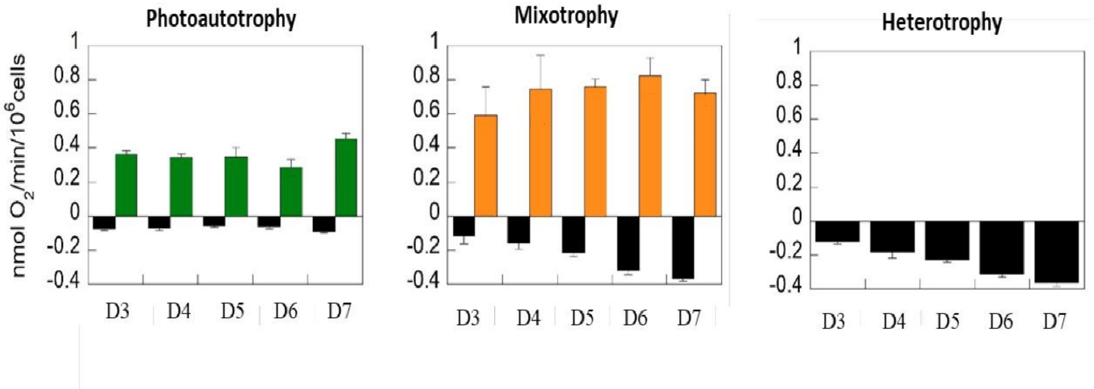

Figure S7: Respiration and net photosynthesis of *G. sulphuraria* SAG21.92 were measured every day in the three different growth conditions (photoautotrophy, mixotrophy and heterotrophy). O_2 consumption or production were measured with a Clark electrode at 42°C. Cells were centrifuged and resuspended at 3 x 10^7 cells in 1 mL of fresh 2xGS medium. Net photosynthesis corresponds to gross photosynthesis +|respiration|. Data from 3 biological replicates ± S.D.

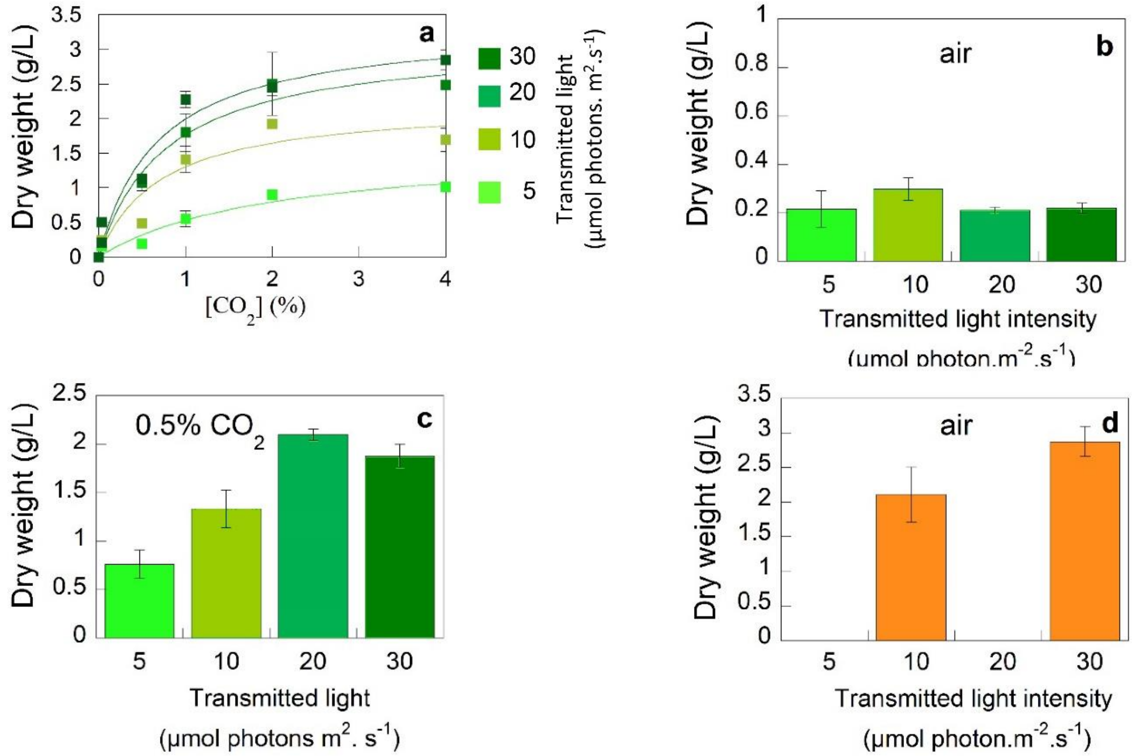

Figure S8: Biomass production of *G. sulphuraria* SAG21.92 as a function of transmitted light intensity and CO_2 concentration. a, Cells were grown in a photobioreactor at four different constant transmitted light intensities and different CO_2 concentrations. During growth, the light intensity was increased to maintain a constant transmitted light (from 5 to 30 µmol photons m^{-2} s^{-1}). Cells exposed to a given light ramp were used to inoculate the next experiment at higher CO_2 concentration, while repeating the same light ramp. Progressive adaptation to higher CO_2 concentration was chosen to avoid acidification of the cytosol in the presence of high CO_2 concentration and to induce a progressive reduction in the accumulation of carbonic anhydrase concentration, which is expected to be high in low CO_2 grown cells. After three days of adaptation, cells were grown for 4 additional days and collected at day 7, dried for three days at 60°C and weighted. Data from 3 biological replicates ± S.D. **b**: Focus on the light dependency of biomass production in phototrophic conditions in air. Same colour code as in panel a. Data from 3 biological replicates ± S.D. **c**, Focus on the light dependency of biomass production in phototrophic conditions supplemented with 0.5% CO_2. Same colour code as in panel a. Data from 3 biological replicates ± S.D. **d**, Focus on the light dependency of biomass production in mixotrophic conditions (sorbitol 25 mM). Two light intensities were tested corresponding to a transmitted light of 10 and 30 µmol photons m^{-2} s^{-1}. Data from 3 biological replicates ± S.D

.

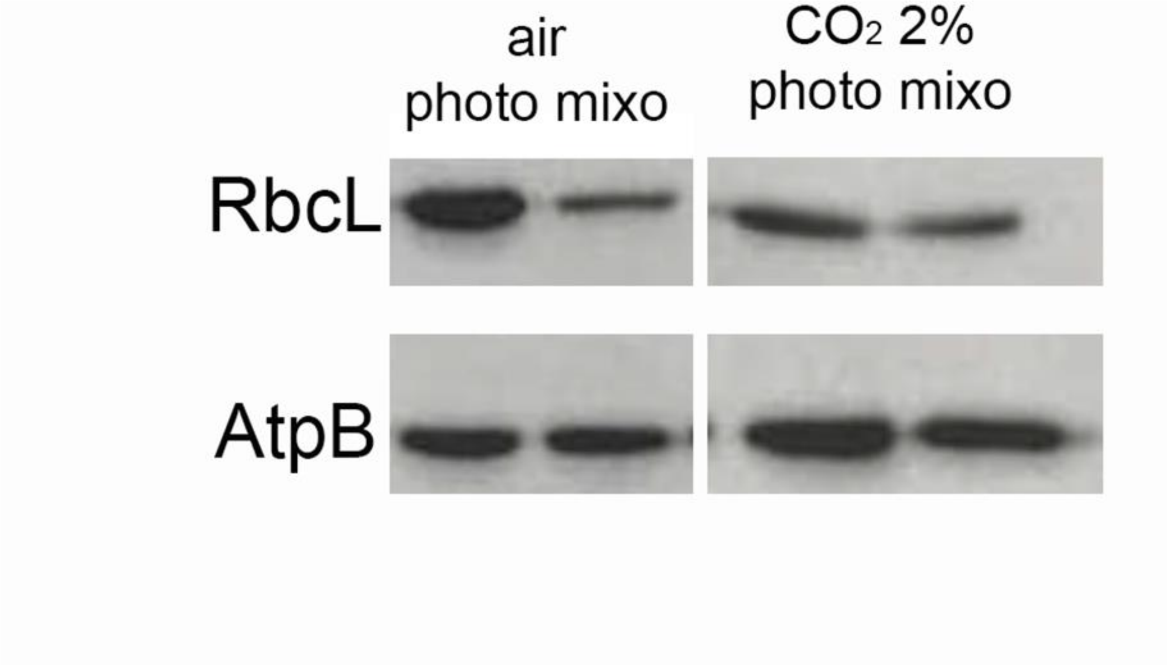

Figure S9: Immunodetection of RuBisCO in phototrophic and mixotrophic cultures of *G. sulphuraria* SAG21.92 under ambient and enhanced CO_2 atmosphere. Cells were grown in a photobioreactor in air ([CO_2]= 0.04 %) or air supplemented with CO_2. After 7 days of adaptation, cells were collected and broken with a Precellys homogeniser, through three cycles of 30 seconds at 10.000 rpm separated by a 30 seconds interval. Total protein extracts were analyzed by immunoblotting with anti- RuBisCO (Agrisera, Sweden). An antibody against the β subunit of the ATP synthase complex (Agrisera, Sweden) was used as a loading control. 10 µg of protein was loaded per well.

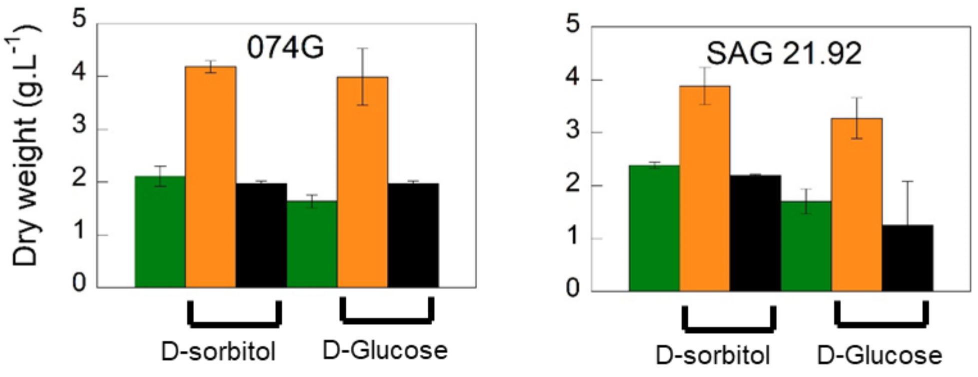

Figure S10: Comparative analysis of phototrophic (green), mixotrophic (orange) and heterotrophic (black) performances in *G. sulphuraria* 074 G and SAG21.92 species with D-sorbitol and D-glucose. *G. sulphuraria* 074 G and SAG21.92 species were grown in a photobioreactor as described in Fig. S3 & S4 under conditions similar to those described in Oesterhelt et al. (2007) in the presence of 2% CO_2 but at 42°C. At day 3 cells were either placed in the dark (black) in the presence of 25 mM D-sorbitol or D-glucose or exposed to light (transmitted light 10 µmol photons m^{-2} s^{-1}) in the absence of organic carbon (green) or in the presence of either 25 mM D-sorbitol or D-glucose as indicated in the graph. Cells were collected at day 7, dried and weighted. Growth performances of the 074G and SAG21.92 strains are very similar in the presence of D-glucose or D-sorbitol. Data from 3 biological replicates ± S.D.

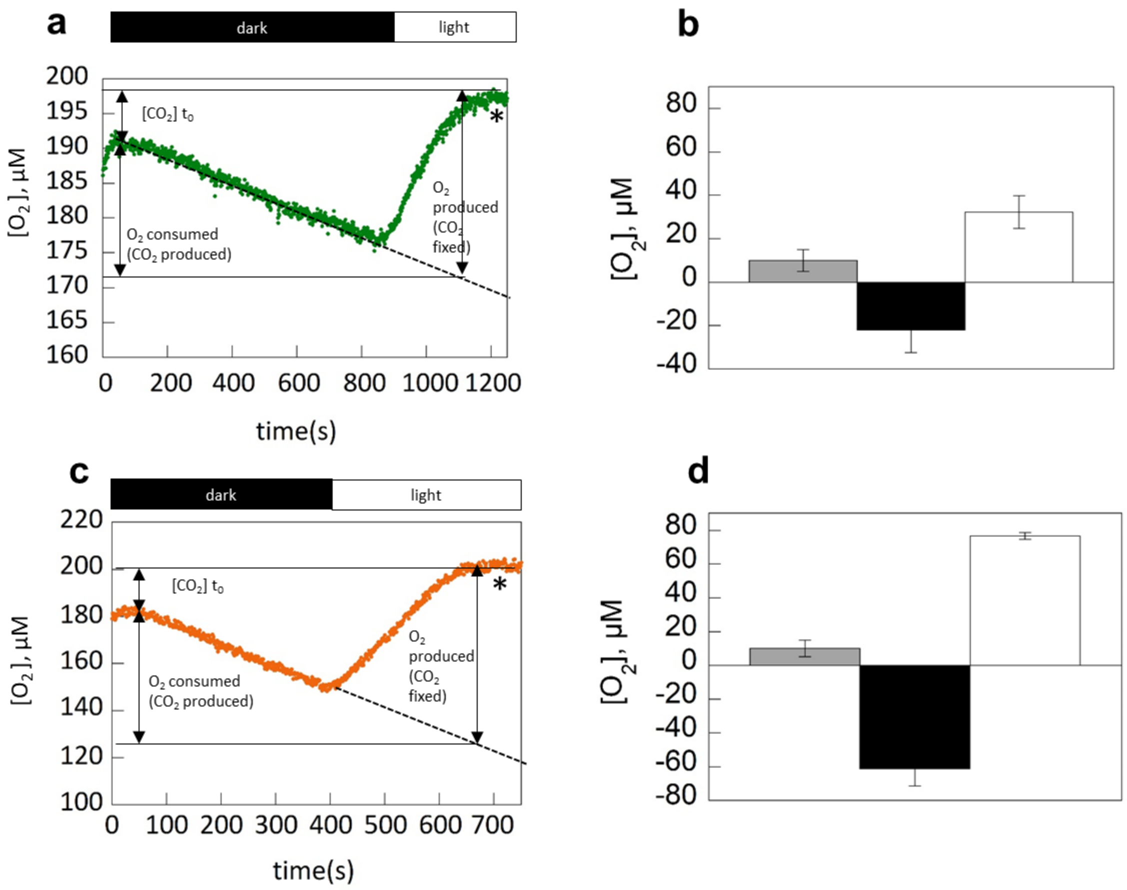

Figure S11: Respiration fuels photosynthesis in photoautotrophic (green) and mixotrophic (orange) *G. sulphuraria* SAG21.92 cultures. **a,c:** representative experiment of O_2 consumption or production measured in a closed system (Clark electrode) at 42°C. Photoautotrophic cells (a,b) or mixotrophic cells (c,d) were centrifuged and resuspended at 30 10^6 cells in 1 mL of fresh 2xGS medium. Stars indicate a dynamic equilibrium reached when all excess CO_2 from the media (CO_2 initially present plus CO_2 produced by respiration) is fixed. Thus, photosynthesis becomes limited by CO_2 production by respiration, leading to the compensation point. **b (photoautotrophic cells) , d (mixotrophic cells)**: the amount of O_2 produced by photosynthesis (white) is commensurate with the amount of CO_2 available to Rubisco, i.e. the sum of respiratory CO_2 (evaluated from O_2 consumption, assuming a 1/1 stoichiometry between CO_2 released and consumed oxygen, black) plus the small CO_2 amount initially present in the medium (10 µM *ca* at pH 2, grey), Data from 3 biological replicates ± S.D.

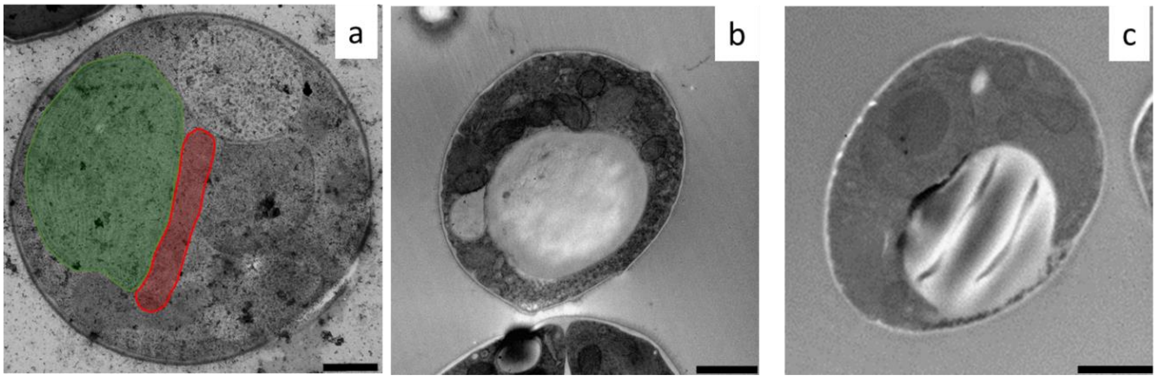

Figure S12: Transmission electron microscopy of *G. sulphuraria* SAG21.92 grown 5 days under photoautotrophic (a), mixotrophic (b) and heterotrophic (c) conditions. b, **c** show the presence of a huge floridean starch grain in the cytosol of the cells four days after addition of 25 mM D-sorbitol. Scale bars: **a**: 0.5 µm; **b**, **c**: 1 µm. In a, mitochondria and plastids are highlighted in red and green, respectively.

Dataset S1 (separate file). Proteins involved in photosynthesis, central metabolism and respiration were selected from the complete proteomic dataset (see Supplementary Dataset S2) and used to build Fig. 3. Proteins function and predicted localizations were checked manually (column "Manual annotation"). Cells highlighted in yellow in this column indicate proteins with statistically significant changes in abundance (see Threshold described in Supplementary Dataset S1). Blue-color and orange-color scales indicate decrease and increase in abundance, respectively (See Legend sheet).

Dataset S2 (separate file). Compared proteomic analysis between photoautotrophic, mixotrophic and heterotrophic growth conditions. Proteomics analyses were performed on biological triplicates for each growth condition. Quantification was obtained from extracting the MS1 intensity. Merging of results and filtering on both identification and quantification criteria as described in the Supporting Information resulted into 2100 proteins ("protein sets", sheet **Dataset S2-ALL**). These 2100 proteins were further divided into 694 proteins entirely missing in at least 1 condition (sheet **Dataset S2-MissingInOneCond.**) and 1406 proteins that have been quantified in all three conditions (in at least 1 replicate, sheet **Dataset S2-SeenInAllConditions**). This latter subset was submitted to differential analysis. Highlighted in blue : imputated values (in Dataset S2-SeenInAllConditions) that were assigned to partially missing abundance values (i.e. within one condition, in Dataset S2-ALL).

Dataset S3 (separate file). Compared metabolomic analysis between photoautotrophic, mixotrophic and heterotrophic growth conditions.
GC-MS sheet: In the table 32 compounds are listed with their retention time and chosen quantifier ion fragment. Compound identification was based on retention time and fragment pattern match with an in-house library or the NIST library. Peak areas of each compound were normalized to the internal standard and transformed to readable values by a multiplicator. The p-value for the sample group was calculated using the multiple comparison function from PRISM software based on

the Students t-test. **IC-MS sheet**: In the table 31 compounds are listed with their retention times and calculated molecular weights. Compound identification was based on 5 different sources of which the in-house database created with mzVault was the preferred source of identification. Batch normalization was carried out with pooled QC samples frequently measured during the batch. The p-value for the sample group was calculated in Compound Discoverer by running the Tukey HSD test (posthoc) after an analysis of variance (ANOVA) test and correction was performed by using the Benjamini-Hochberg algorithm for the false discovery rate.

Supporting information references

Allen GJ. 1959. Studies with *Cyanidium caldarium*, an anomalously pigmented chlorophyte. *Archiv für Mikrobiologie* **32**: S. 270-277.

Bouyssie D, Hesse AM, Mouton-Barbosa E, Rompais M, Macron C, Carapito C, de Peredo AG, Coute Y, Dupierris V, Burel A, et al. 2020. Proline: an efficient and user-friendly software suite for large-scale proteomics. *Bioinformatics* **36**(10): 3148-3155.

Cuaresma M, Janssen M, van den End EJ, Vilchez C, Wijffels RH. 2011. Luminostat operation: A tool to maximize microalgae photosynthetic efficiency in photobioreactors during the daily light cycle? *Bioresource Technol* **102**(17): 7871-7878.

Huber W, Von Heydebreck A, Sultmann H, Poustka A, Vingron M. 2002. Variance stabilization applied to microarray data calibration and to the quantification of differential expression. *Bioinformatics* **18**(Suppl 1): S96-104.

Johnson X, Vandystadt G, Bujaldon S, Wollman FA, Dubois R, Roussel P, Alric J, Beal D. 2009. A new setup for *in vivo* fluorescence imaging of photosynthetic activity. *Photosynthesis Research* **102**(1): 85-93.

Maxwell K, Johnson GN. 2000. Chlorophyll fluorescence - a practical guide. *Journal of Experimental Botany* **51**(345): 659-668.

Oesterhelt C, Schmalzlin E, Schmitt JM, Lokstein H. 2007. Regulation of photosynthesis in the unicellular acidophilic red alga *Galdieria sulphuraria. Plant Journal* **51**(3): 500-511.

Wieczorek S, Combes F, Borges H, Burger T. 2019. Protein-level statistical analysis of quantitative label-free proteomics data with ProStaR. *Methods in Molecular Biology* **1959**: 225-246.

Wieczorek S, Combes F, Lazar C, Gianetto QG, Gatto L, Dorffer A, Hesse AM, Coute Y, Ferro M, Bruley C, et al. 2017. DAPAR & ProStaR: software to perform statistical analyses in quantitative discovery proteomics. *Bioinformatics* **33**(1): 135-136.
